# Vegetation Analysis of Wetland Ecosystems in Southern Turkey Using the Fuzzy Means Method

**DOI:** 10.3390/biology14060710

**Published:** 2025-06-17

**Authors:** Deniz Boz

**Affiliations:** 1 Department of Biology, Faculty of Arts and Sciences, Cukurova University, Adana 01250, Turkey; denizkar@cu.edu.tr; 2 Department of Tourism Management, Kozan Faculty of Business, Cukurova University, Adana 01250, Turkey

**Keywords:** Göksu Delta, wetland ecosystem, SEPA, fuzzy means, vegetation ecology, wetland habitat, coastal zone

## Abstract

In this study, the vegetation of the natural area of the Göksu Delta Special Environmental Protection Agency (SEPA), one of Turkey’s most important wetlands, is researched. The importance of this study in terms of contributing to environmental protection and land use planning studies reveals that this natural area, where rare ecosystems are found, has started to degrade and disappear under human influence. This study was conducted because the area is not only a designated RAMSAR wetland (a wetland site designated of international importance especially for the Waterfowl Habitat under the Ramsar Convention) but also includes nearby residential developments. With this study, the vegetation of the area was studied to determine the syntaxonomic units across different habitats. The natural area of Göksu Delta is divided into three main habitat groups: aquatic, sand dune, and halophytic. In the research, the Braun-Blanquet method was used. During the research in the Göksu Delta, 279 sample areas were surveyed. The data were analysed according to the fuzzy means cluster method. During the investigation, 29 associations were identified, and 16 of them are considered a new finding for science. These 29 associations can be classified as follows: aquatic vegetation is represented with four associations (three of them belong to *Phragmito-Magnocaricetea* and one of them belongs to Potametea classes), sand dune vegetation is represented with 12 associations (belonging to Ammophiletea Br.-Bl. & Tüxen ex Westhoff, Dijk, & Passchier 1946 class), and halophytic vegetation is represented with 13 associations (six of them belong to Salicornietea fruticosae Br.-Bl. & Tüxen ex A. & O. Bolòs 1950, six of them belong to Juncetea maritimi Br.-Bl. in Br.-Bl., Roussine & Nègre 1952, and one of them belong to Molinio-Juncetea Br.-Bl. (1931) 1947 classes). Three (*Onopordum boissieri*, *Ambrosia maritima,* and *Chlamydophora tridentata*) of the endemics and rare plants that were explored during the study were recorded as new alliance characteristics.

## Simple Summary

In this study, the vegetation of the Göksu Delta Special Environmental Protection Area, one of Turkey’s most important wetlands, was investigated. The delta natural area is divided into three main habitat groups: aquatic, sand dune, and halophytic. During research in the delta, 279 samples were examined, and 29 associations were identified, 16 of which are new to science. Of these, aquatic vegetation is represented by 4 associations, sand dune vegetation by 12 associations, and halophytic vegetation by 13 associations. Three of the endemic and rare plants discovered during the study were recorded as new alliance characteristics. The delta is an international RAMSAR site. The delta, one of the most important wetlands in the Middle East and Europe, is one of Turkey’s most important sand dune areas, a habitat for halophytic plants, an important bird and plant area, and an important breeding ground for turtles. This research will form the basis for environmental protection and land use planning studies in the natural areas of the Göksu Delta Special Environmental Protection Area. This research is important in terms of protecting the region from environmental pollution and ecological degradation, and ensuring that natural resources and historical remains are preserved for future generations.

## 1. Introduction

The Göksu Delta SEPA is one of the most important RAMSAR wetlands in the Middle East and Europe. SEPA, as an obligation imposed by the Convention for the Protection of the Mediterranean Sea against Pollution (Barcelona), these are the areas that are of ecological importance in our country and the world and are under special protection by the Decree of the Council of Ministers because they are at risk of deterioration or extinction due to pressures such as industry, tourism, and construction. The research area is a turtle breeding zone, a habitat of halophytic plants, and one of Turkey’s most significant sand dunes. For example, drainage and watering channels are important habitats for aquatic plants in small and large salty and freshwater lakes, such as Akgöl and Paradeniz. Therefore, the research area includes SEPA, the RAMSAR wetland, the Key Biodiversity Area, Important Bird Area, Important Plant Area, Wildlife Protection and Improvement Area, Sensitive Zone, and the First Degree Natural Site Area.

In terms of plant geography, Göksu Delta generally belongs to the Tethys sub-kingdom, which is a border and migration area of Boreal and Tropical floras, which belong to the Holarctic kingdom [1]. Göksu Delta is located in the East Mediterranean subregion, part of the Mediterranean region, one of the four regions of the Tethys sub-kingdom [1]. Since the Göksu Delta is located on the coastline, there is a lot of anthropogenic pressure and destruction.

Despite the forest vegetation of the Mediterranean region being very well studied, aquatic, sand dune, and halophytic vegetation in its wetlands are not well-investigated [2]. From 1977 to 1999, the flora and vegetation of some areas in the Göksu Delta SEPA were already studied. According to the literature, there are three articles on the aquatic, sand dune, and halophytic vegetation in the Göksu Delta SEPA area. These studies, such as those by Uslu (1977) [3], Gehu et al. (1989) [4], and Gürkan et al. (1999) [5], are considered important ones. The following works of literature were used for the nomenclature of the associations and syntaxonomic units [6]. Although the dominant species are the same in these three articles, the associations with different floristic composition, soil, and water characteristics are named as new. These studies on the vegetation of the research area can be listed as follows: Bekat and Seçmen (1984, 1988) [7,8], Çakan et al. (2003) [9], Çetik (1982) [10], Gehu and Uslu (1989) [11], Hamzaoğlu and Aksoy (2006) [12], Kavak (2006) [13], Kılınç et al. (1992) [14], Kılınç and Karaer (1995) [15], Küçüködük (1987) [16], Öner et al. (1973a,b) [17,18], Özkanca (1989) [19], Özen and Kılınç (1995) [20], Seçmen (1977) [21], Seçmen and Leblebici (1978, 1980, 1982, 1984, 1987, 1991, 1992, 1994, 1996) [22,23,24,25,26,27,28,29,30], Seçmen et al. (1986) [31], Yurdakulol (1974) [32], Yurdakulol et al. (1996) [33], Rivas-Martinez (1997) [34], Rivas-Martinez et al. (1999, 2001) [35,36]. Coastal sand dunes are the most threatened ecosystems. In light of the limited number of vegetation and habitat classification studies that have employed the fuzzy means method, this study stands to make a significant contribution to the extant literature [37,38].

In this study, the vegetation of the Göksu Delta SEPA is investigated. The vegetation study was carried out according to the sample area method of Braun-Blanquet [39]. Göksu Delta SEPA is categorised within the NATURA2000 habitat classification, which subdivides the area into three distinct groups: aquatic, dune, and halophytic. The fuzzy means method was utilised to calculate the similarity percentages of water and soil samples, and habitat subgroups were determined [40]. At the end of the study, the plant associations, which spread to different habitats in the area, and their syntaxonomic upper categories were determined.

Given the significance of this study area as a RAMSAR wetland and its current state of anthropogenic disruption, primarily characterised by human settlements, it has been hypothesised that the consequences of the research will contribute significantly to the advancement of environmental protection and land use planning studies.

## 2. Materials and Methods

**Figure 1:**
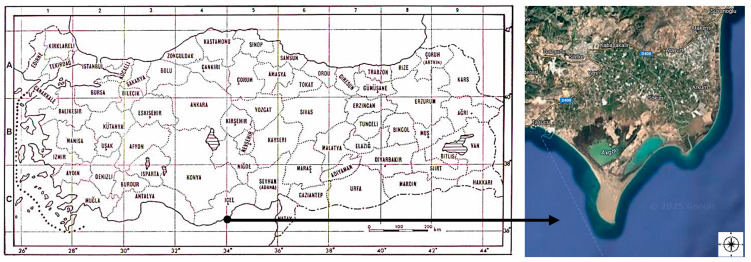
Location of the research area in the Turkey grid system map and a satellite image of the research area [45].

The research area covers the natural habitats of Göksu Delta SEPA. The area is located in the C4 and C5 grids of Davis (Figure 1) [41]. The altitude of the area varies between 0 and 8 m. The Göksu Delta is formed by sediments carried by the Göksu River, 150 km^2^ (relatively without tide) and a delta type that extends to the sea. The west and east coasts of the delta are affected by water coming from the Mediterranean Sea. The research area has permanent water masses, namely, the Paradeniz Lagoon, Kuğu Lake, Ak Lake, and Arapalanı Lake. The geological structures in the research area mostly consist of clay, sand, and gravel. Under normal conditions, there is a lot of sediment coming from the Göksu River; however, the amounts brought by winds and streams are very small [5]. The morphology of the delta is shaped by the changes in the flow direction of the river, winds, sea streams, and strong winds. The strongly blowing winds are the main cause of sand dune formations at the coastline [11]. The research area is still undergoing changing geomorphologic processes. The area has various soil types: Xerochrept, Haloquept, Xerofluvent, and Fluvaquent [42]. Alluvial soil is found throughout most of the delta. The research area is located in the semi-arid soft Mediterranean bioclimatic region according to the Emberger method [43,44].

Habitats in the research area are classified according to the NATURA2000 criteria [46,47]. To determine the vegetation in different habitats, the Braun-Blanquet (1964) method was used [48]. The sampling areas in different habitats were obtained using the “minimal sample area” method of Braun-Blanquet (1964), considering the principle of the homogeneity of the dominant species, floristic composition, and ecology. The sample areas were determined as 25 and 100 m^2^ in aquatic habitats, 16 and 50 m^2^ in dune habitats, and 9 and 100 m^2^ in halophyte habitats. Instead of the Braun-Blanquet cover-abundance scale, the Barkman cover-abundance scale was utilised, as it is a more detailed scale and may provide more information for comparing sample areas [49].

Soil and water samples representing various associations across different seasons were collected during sampling studies conducted in 2004 and 2005. Soil samples, which were collected from the area, were analysed according to the following 12 variables: saturation%, total salt%, pH value, lime%, useful P_2_O_5_, organic matter, texture analysis, colour value, K_2_O, sand%, clay%, and silt%. Among the analyses, saturation% was determined by saturating the soil with distilled water and expressing the result as%. Total salt% was determined by using the Saikl Survey Staff (1951) chart by measuring the electrical conductivity in water-saturated soil with a conductivity instrument [50]. pH was determined by measuring with a digital pH meter. Lime% was determined by the Volumetric Calcimetry method. Organic matter was determined by the Walkley–Black method of Richards (1954) [50]. Beneficial P_2_O_5_ was determined from the standard scale via spectrophotometer readings using the method of Olsen et al. Beneficial K_2_O_5_ analysis was determined by Flame Photometer readings using the method of Richards (1954) [51]. Texture analysis was performed using the hydrometer method [52]. The sand%, clay%, and silt% were calculated using the results obtained. In order to determine the texture fractions in the sandy samples, sand sizing was performed using sieve analysis with 17 different particle sizes between 75,000 µ and 74 µ. The results of this analysis were evaluated according to the texture fraction scale made according to the German system [53]. According to this scale, sand sizes were divided into three groups as coarse, medium, and fine. In different seasons, water samples were collected from the temporary and permanent wetlands in the research area. On the other hand, 19 different analyses, which are colour–taste–odour value, amount of sediment, pH value, conductivity (25 °C), salt, NA, Ca^+2^ + Mg^+2^, Ca, Mg, total cation, CO_3_, HCO_3_, Cl, total anion, Na%, SAR, and RSC were carried out on the water samples collected in the research area. As a result of these analyses, the seasonal water changes of each locality were revealed. Among the physical analyses, colour was determined by using colour darkness standards prepared with a Hexachloraplatinate-Cobalt chloride solution. The odour test was performed by sense of smell immediately after sampling. The amount of sediment and turbidity was determined by looking at the condition of the sample. Among the chemical analyses, pH was measured with a digital pH meter. EC (25 °C), which is a conductivity analysis, was calculated by reading with the electrode of the conductivity device. Total salt content was calculated using the value read by the conductance device. Total hardness determination (Ca^+2^ + Mg^+2^) was performed using an Eriochramblack indicator, Ca^+2^ determination was performed using a Murexide and salt indicator, Cl_2_ determination was performed using a Potassium Chromate indicator, HCO_3_ determination was performed using a methyl orange indicator, and Nitrite determination was performed using a Trammosdorf indicator. CO_3_ determination was performed in samples with a pH value greater than 8, and a Phenolphthalein indicator was used. As a result of all these analyses, Cation sum, Anion sum, Mg, Na and Na% determination, and SAR and RSC calculations were made using the values obtained.

In the floristic evaluation of syntaxa, the following features were used: characteristics and distinctive species, dominance, frequently existing species, endemicity, threatened categories, life duration, structure, life form, floristic element, and availability. The ones that are determinative and descriptive for the association were selected and evaluated. The presence of the species forming the identified associations was found by calculating the % frequency with the Braun-Blanquet presence-constancy five-grade scale [54]. Accordingly, constant and rare species were identified in sampling areas. The identified syntaxa were then classified as similar or dissimilar to the syntaxa in other researchers’ studies. The similar ones were classified in chronological order. The dissimilar ones were identified geographically by searching first the places closest to the study area, then distant places and finally other countries. The floristic, habitat, and ecological characteristics of these new associations were compared with other associations. The similarity ratios of the new associations with other associations were calculated according to the “Sorenson similarity index”. The floristic similarity rates of these new associations ranged from 2.5% to 44.4%. Although some of the associations have high similarity rates, their characteristic and distinctive species are different, and only the dominant species are similar.

The syntaxonomic categories of the associations were determined according to Rivas-Martinez (1997) [34], Rivas-Martinez et al. (1999, 2001) [35,36], Braun-Blanquet et al. (1951) [39], and the Fransa Tela Botanica group (Assosiation, 2000) [55]. The similarity percentages of the features of floristic, soil, and water sampling areas were calculated according to three clustered methods in the fuzzy means, since there are three main habitat groups (aquatic, sand dune, and halophytic). The associations were determined both according to the result of the fuzzy means calculations and the ecological data collected from the area. Those associations obtained from the research, which have floristic and ecological differences compared to the associations listed in the literature, are considered new and presented. The new associations of the research were named according to rules indicated in the “International Code of Phytosociological Nomenclature” [56].

## 3. Results and Discussion

Göksu Delta SEPA is divided into three main habitat groups, which are aquatic, sand dune, and halophytic, according to the NATURA2000 habitat classification. These main habitat groups were also divided into 12 subgroups, of which 279 sample areas were surveyed. According to the kind of habitat, water and/or soil samples were collected from the sample areas where the dominating species are different. Based on the above-mentioned 12 soil analyses, similarity percentages and cluster groups were determined using the fuzzy means method according to the kind of habitat (Table 1). The fuzzy means method was utilised as a support tool for the NATURA2000 classification. The habitat groups, as determined by the NATURA2000 classification, were then overlapped with the fuzzy mean method triple clustering method. It was determined that the associations observed within these habitats were consistent with those established through the Braun-Blanquet method.

Similar to soil analysis, similarity percentages and cluster groups were determined after completing the 19 different analyses on water samples (Table 2).

According to the Braun-Blanquet method, the tables of sampling areas were prepared while in the field. After completing the analysis, the associations and syntaxonomic units were determined by taking into consideration ecological appraisals and literature data. In the final stage, 29 associations were determined where aquatic, sand dunes, and halophytic vegetation were represented by 4, 12, and 13 associations, respectively. The outcome of the research on vegetation can be seen from Table A1 given in the paper in Appendix A.

### 3.1. Aquatic Vegetation

In the aquatic habitats of the research area, 40 sampling areas were surveyed (Table 3). Consequently, four associations were determined in this vegetation, as one can see from Table A1.

**Figure 2:**
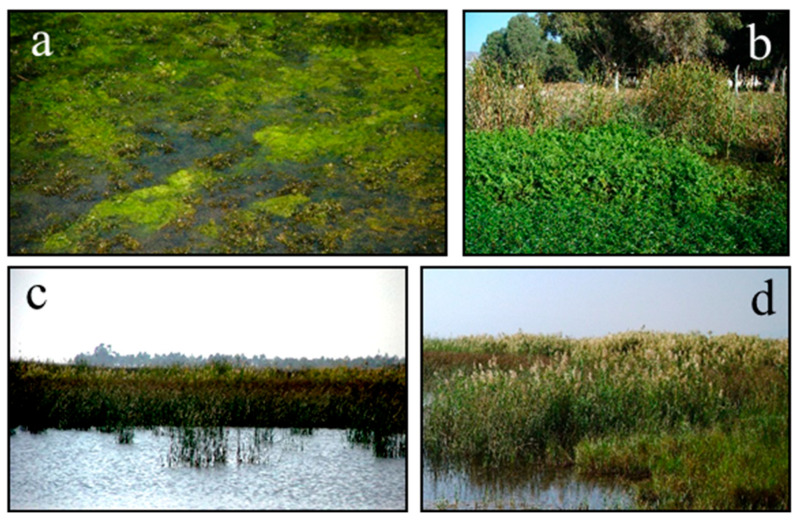
General view of the associations in the aquatic vegetation: (**a**) *Potamogetono pectinati-Ceratophylletum demersi* (Hild & Rehnelt 1965) Passarge 1995, (**b**) *Ludwigio stoloniferae-Nasturtietum officinalis* ass. nov., (**c**) *Ruppio cirrhosae-Schoenoplectetum littoralis* ass. nov., (**d**) *Bolboscoeno maritimi* var. *cymos-Pragmitetum australis* Borhidi & Balog 1970.

*Potamogetono pectinati-Ceratophylletum demersi* (Hild & Rehnelt 1965) Passarge 1995, one of the associations detected in the in-channel habitats, is located in places where the water is deeper with a strong current than the other association detected in the canal (Figure 2a). Furthermore, the second association, *Ludwigio stoloniferae-Nasturtietum officinalis* ass. nov., is located in areas less exposed to water movement, where the depth is less (Figure 2b). *Ludwigio stoloniferae-Nasturtietum officinalis* ass. nov. has a relatively high dominant coverage rate, which varies between 80 and 100%. According to the literature review, it has been observed that the association, which consists of the dominant species *Nasturtium officinale*, has very different floristic and ecological compositions. That is why the similarity between the literature and the field study is only the existence of dominant species. In the in-lake vegetation, *Ruppio cirrhosae-Schoenoplectetum littoralis* ass. nov. association is an association (Figure 2c) that occurs only in the in-lake vegetation at depths up to 80 cm, in places with slightly basic pH, medium–high saline water (brackish), not rich in plant diversity, and 86% of which are geophytes. This association can only be seen in the lakes in which the variety of floristic composition is not so rich, of which 86% belongs to geophytes. Because of the features mentioned above, this association is different from the other associations in which dominant species are the same [9,30]. The dominant species of the *Bolboscoeno maritimi* var. *cymos-Pragmitetum australis* Borhidi & Balog 1970 association (*Phragmites australis*), which occurs in river and lakeside vegetation, is a Euro-Siberian floristic element common in aquatic habitats (Figure 2d).

### 3.2. Sand Dunes Vegetation

**Figure 3:**
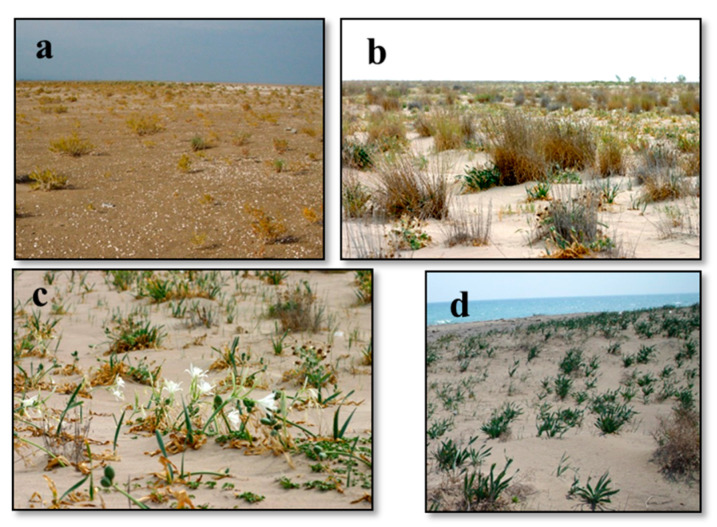
General view of the associations in sand dune vegetation I: (**a**) *Cakilo maritimae-Zygophylletum albi* Çakan, Düzenli, & Karaömerlioğlu 2003, (**b**) *Cypero mucronati-Agropyretum juncei* Kühnholtz ex Br.-Bl. 1933, (**c**) *Eryngio maritimi-Pancratietum maritimi* Çakan, Düzenli, & Karaömerlioğlu 2003, (**d**) *Ambrosio maritimae-Pancratietum maritimi* ass. nov.

In the sand dune of the research area, 89 sampling areas were surveyed, and these sampling areas were grouped into 12 associations (Table 4). Nearly half of the taxa, which take place in this vegetation, are Mediterranean floristic elements. In most of the associations in the dune vegetation, endemic and rare plants are present as dominant, characteristic-distinctive, or associated species with more or less abundance. In this research, *Ambrosia maritima* (threatened categories EN) and endemic *Onopordum boissieri* (threatened categories LR(nt)) species are described as characteristic of the alliance involving Euphorbio-Ammophilion arundinaceae Br.-Bl. (1931) 1933 em. J.M. et J. Gehu 1988. The names of rare plant species and their threatened categories are as follows: *Hypericum polyphyllum* subsp. *polyphyllum* LR(nt), *Arum dioscoridis* var. *dioscoridis* VU, *Bromus psammophilus* CR, *Cyprina gracilis* EN, *Zygophyllum album* VU, *Pancratium maritimum* EN, *Limonium graecum* var. *graecum*, and *Alhagi manniferae* VU. *Cakilo maritimae-Zygophylletum albi* Çakan, Düzenli, & Karaömerlioğlu 2003, *Cypero mucronati-Agropyretum juncei* Kühnholtz ex Br.-Bl. 1933, *Eryngio maritimi-Pancratietum maritimi* Çakan, Düzenli, & Karaömerlioğlu 2003 and *Ambrosio maritimae-Pancratietum maritimi* ass. nov. associations were identified on the shifting coastal dunes vegetation (Figure 3a–c). The distinctive feature of the dominant-characteristic-distinctive species of the *Ambrosio maritimae-Pancratietum maritimi* ass. nov. association is that they are found in the EN endangered category on the northern and southern coasts of Turkey, and at the same time, their cover and abundance are high (Figure 3d). In addition, although the characteristic classes of these associations are the same (Ammophiletea), there are many representatives of the Stellerietea media class in this association. This association is common on protected plains that exist behind the shifting dune hills. It was noticed that floristic and ecological similarities between the association and the others in which dominant species are the same are low [3,9,15].

**Figure 4:**
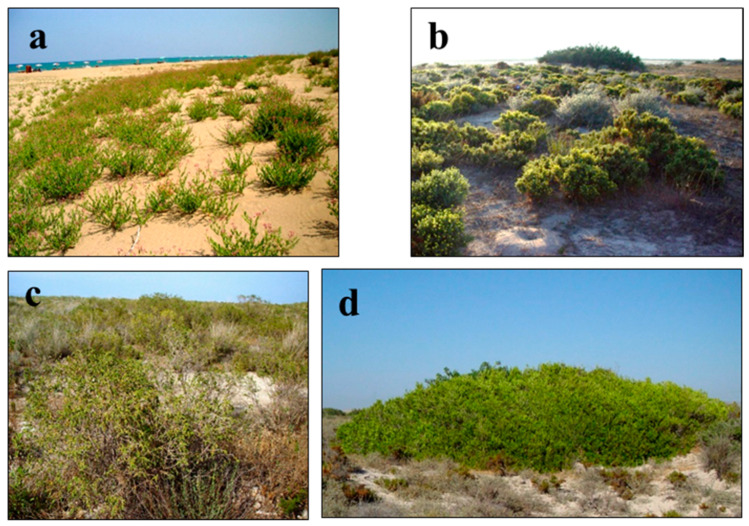
General view of the associations in sand dune vegetation II: (**a**) *Cypero capitati-Trachomitetum veneti* ssp. *sarmatiense* ass. nov., (**b**) *Echio angustifolii-Ononidetum natrix* ssp. *hispanicae* ass. nov., (**c**) *Parapholido incurvae-Thymelaeetum hirsutae* ass. nov., (**d**) *Sorgho halepense* var. *halepense-Myrtetum communis* ssp. *communis* ass. nov.

In fixed or semi-shifting sand dune vegetation, *Cypero capitati-Trachomitetum veneti* ssp. *sarmatiense* ass. nov. association’s soils are slightly salty, and since it is under the influence of wind, its quantity of organic material is low (Figure 4a). The dominant species in this association is scrub, under which there is a rich herb layer with 80% coverage. The floristic and ecological similarities between the association and the others in which the dominant species are the same are low [9]. The *Echio angustifolii-Ononidetum natrix* ssp. *hispanicae* ass. nov. association exists on the sand dune hills and plains, which are 1.5 and 2 m in height (Figure 4b). The associations that belong to this vegetation prefer basic soil the most. It was noticed that floristic and ecological similarities between the association and the others in which the dominant species are the same are low [3]. *Parapholido incurvae-Thymelaeetum hirsutae* ass. nov. association is identified on the flats as high as 80 cm, which is far from the effect of the sea (Figure 4c). It was noticed that the soil is sandy; however, it has the lowest sand percentage with respect to the other associations. In the association, dominant class characteristics exist, and Salicornieteae fruticosae and Stellerietea media class characteristics, where saltiness is high, are also seen. It was noticed that floristic and ecological similarities between the association and the others in which dominant species are the same are low [9].

*Sorgho halepense* var. *halepense-Myrtetum communis* ssp. *communis* ass. nov. association is identified abundantly in sand dune shrub vegetation (Figure 4d). This association is distributed with a high abundance and cover (85–100%), especially on sand dunes under the influence of moist winds coming from the sea. It was noticed that floristic and ecological similarities between the association and the others in which dominated species are the same are extremely low (Table 5).

**Figure 5:**
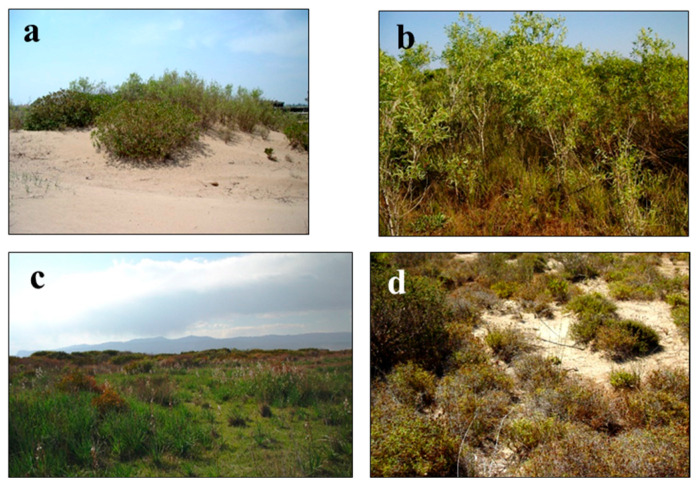
General view of the associations in sand dune vegetation III: (**a**) *Polygono equisetiformis-Viticetum agni-casti* ass. nov. (**b**) *Ephedro campylopodae-Populetum euphraticae* ass. nov., (**c**) *Urgino maritimi-Asphodeletum aestivi* ass. nov., (**d**) *Verbasco sinuati-Sarcopoterietum spinosi* ass. nov.

In the research area concerning *Polygono equisetiformis-Viticetum agni-casti* ass. nov., an association was identified not only on the low flats but also on the highest (8 m) sand dunes (Figure 5a). These hills are different from other associations in that they possess tiny sands and an abundance of organic materials. Coverage is as high as 80 to 100%, and *Populus euphratica* constitutes the tree layer (Figure 5b). The syntax of this association is not found in previous research. The soil structure of damaged sand dune vegetation is sandy, and its size is thin and medium. The coverage of associations is as high as 95 to 100%.

The *Urgino maritimi-Asphodeletum aestivi* ass. nov. association is located in the areas damaged by fires (Figure 5c). It is constituted as an herb layer. The floristic composition of the area is very rich, and the number of annual plants is high, as the area offers many annual plants the opportunity to live after fire destruction. This pattern has enhanced the concentration of organic materials in the association, which extends to soils. The syntaxa of this association is not found in previous research. According to the *Urgino maritimi-Asphodeletum aestivi* ass. nov. association, the *Verbasco sinuati-Sarcopoterietum spinosi* ass. nov. association extends on less humid and more sandy soils (Figure 5d). It was noticed that floristic and ecological similarities between the association and the others in which the dominant species are the same are low [3,4,7,8,15,22].

### 3.3. Halophytic Vegetation

**Figure 6:**
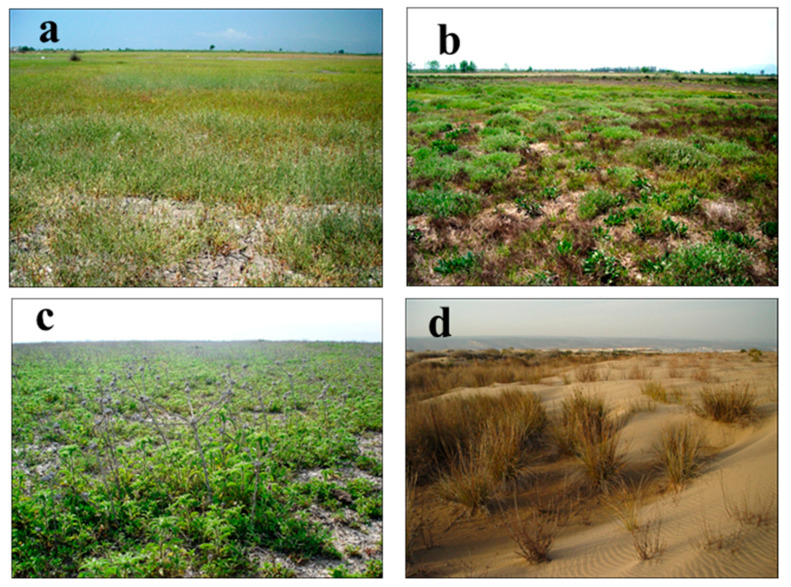
General view of the associations in the halophytic vegetation I: (**a**) *Limonio gmelinii-Aeluropetum littoralis* (Bab. 1979) Gehu & Uslu 1989, (**b**) *Limonio angustifolii-Halimionetum portulacoides* ass. nov., (**c**) *Asterisco aquaticae-Plantaginetum coronopi* ssp. *commutati* ass. nov., (**d**) *Polypogon maritimus* ssp. *maritimus-Juncetum littoralis* Çakan, Düzenli, & Karaömerlioğlu 2003.

A total of 150 units of sampling area were surveyed on the halophytic vegetation of the research area. According to the analysis, 13 associations have been identified (Table 6). This vegetation grows on the clay and silt soils. As the concentration of salt in the soil changed, it was noticed that the spread of dominant species was also changing. Taxa of *Chlamydophora tridentata* (VU), *Salicornion fruticosae* Br.-Bl. 1933, and *Ambrosia maritima* (EN) are identified as *Euphorbio-Ammophilion arundinaceae* Br.-Bl. (1931) 1933 em. J.M. et J. Gehu 1988 alliance characteristics. The soil structure of *Limonio gmelinii*-*Aeluropetum littoralis* (Bab. 1979) Gehu & Uslu 1989 association is a tiny fraction of sand and a moderate level of salt (Figure 6a). *Limonio angustifolii-Halimionetum portulacoides* ass. nov. association is quite widespread in the research area (Figure 6b). The soils of these areas contain more or less salt, silt, and clay and a high content of organic material. It was noticed that there is no association in the literature in which floristic and ecological features are significantly similar to the findings of the association in the research area [9,33]. *Asterisco aquaticae-Plantaginetum coronopi* ssp. *commutati* ass. nov. association is widespread on little salty thin sandy flatness, which had been destroyed a long time ago (Figure 6c). General coverage is as high as 90 to 100%, where herbal vegetation is abundant. The vegetation of saline temporary ponds, dominated by members of the genus *Juncus*, is moist, with flooded depressions or plains with fresh or saline water flooding from the bottom. It is found to possess Euro-Siberian floristic elements, where there is a decrease in salinity in the water and Mediterranean floristic elements, where salinity is increased.

**Figure 7:**
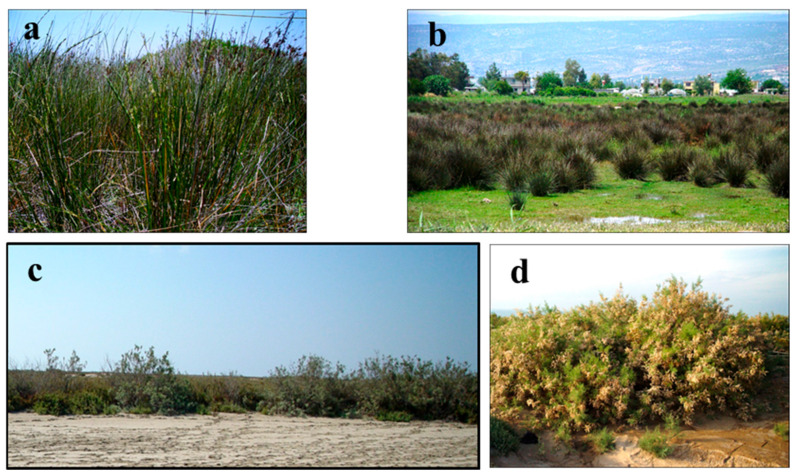
General view of the associations in the halophytic vegetation II: (**a**) *Phragmiti australis-Juncetum maritimi* Vural, Duman, & al. 1995, (**b**) *Atriplici hastatae-Juncetum acuti* Çakan, Düzenli, & Karaömerlioğlu 2003, (**c**) *Arthrocnemo fruticosii-Tamaricetum tetragynae* ass. nov., (**d**) *Salicornio fragilis-Tamaricetum tetrandrae* ass. nov.

*Polypogon maritimus* ssp. *maritimus-Juncetum littoralis* Çakan, Düzenli, & Karaömerlioğlu 2003 association is widespread, especially in humid depressions or freshwater inflow of areas (Figure 6d). *Phragmiti australis-Juncetum maritimi* Vural, Duman, & al. 1995 association is located in high halophytic depressions and the plains of groundwater (Figure 7a). *Atriplici hastatae-Juncetum acuti* Çakan, Düzenli, & Karaömerlioğlu 2003. Unlike the other associations of this vegetation, it is located in low-salinity or salt-free high groundwater and flooded areas (Figure 7b).

The vegetation of saline temporarily flooded plains is dominated by members of the genus, *Tamarix*. These areas may have a water depth of ~25–35 cm from time to time. This vegetation is generally clayey and silty, with light–moderate alkaline, very calcareous, and moderately saline soils. The *Tamaricetum smyrensis* Seçmen & Leblebici 1996 association has helped to identify flooded areas around lakes and ponds in the area (Table 7). *Arthrocnemo fruticosii-Tamaricetum tetragynae* ass. nov. association is widespread close to watery swamps, floodplains, and channels (Figure 7c). The dominant tall shrub layer is characterised by vegetation abundance and more (80–100%), and contains a herb layer that is rich underneath. There is no similarity between this unit and any association or community other than the dominant type of partnership.

*Salicornio fragilis-Tamaricetum tetrandrae* ass. nov. association is distributed in watery marshes or flooded areas (Figure 7d). General overlap is also very high (90–100%), and the floristic composition is very rich. Characteristics include *Tamarix tetrandra*, the dominant shrub, *Salicornia fragilis*, and *Arthrocnemum glaucum*, with a high abundance and cover in its lower layers. This association has low floristic and ecological similarity with other associations with the same dominant species [12].

**Figure 8:**
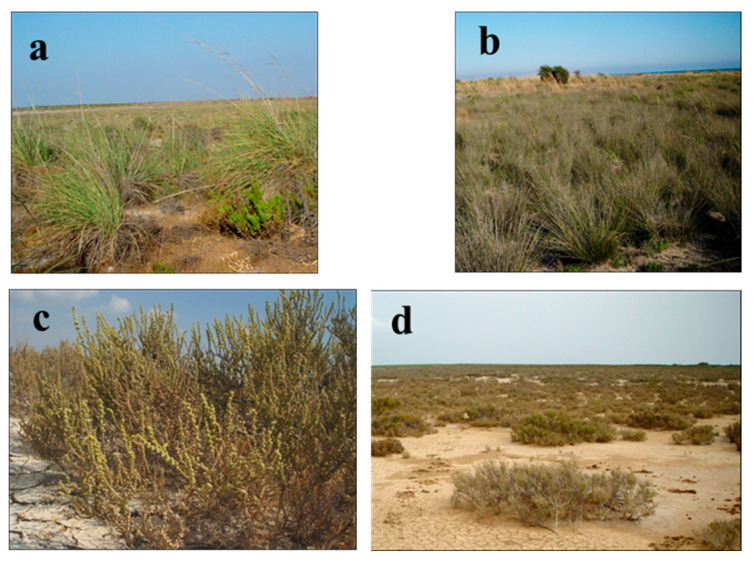
General view of the associations in the halophytic vegetation III: (**a**) *Schoeno nigricantis-Saccharetum ravennae* Çakan, Düzenli, & Karaömerlioğlu 2003, (**b**) *Eriantho-Schoenotum nigricantis* (Pign. 1953) Gehu 1984, (**c**) *Arthrocnemo-Halocnemetum strobilaceii* Oberd 1952, (**d**) *Salicornio europaeae-Arthrocnemetum fruticosum* Çakan, Düzenli, & Karaömerlioğlu 2003.

The floristic composition of the *Schoeno nigricantis-Saccharetum ravennae* Çakan, Düzenli, & Karaömerlioğlu 2003 association is rich, and the overlap is high (90–100%) (Figure 8a). Unlike other saline habitats, regarding *Eriantho-Schoenotum nigricantis* (Pign. 1953) Gehu 1984, the soils in which the association is distributed are more sandy, 43% of which are coarse fractionated (Figure 8b). Almost half of the flora of the association consists of perennial grasses. Saline terrestrial plains are located further inland from the coast, away from the coastal influence, and have high clay and silt content.

There is a visible accumulation of salt on the surface of these areas. In this vegetation (Table 8), on the saltiest soils with low levels of available phosphorus and potassium, the *Arthrocnemo-Halocnemetum strobilaceii* Oberd 1952 association is found (Figure 8c). The association *Salicornio europaeae-Arthrocnemetum fruticosum* Çakan, Düzenli, & Karaömerlioğlu 2003 is distributed in very saline wet-humid plains in very large areas (Figure 8d).

In the Göksu Delta SEPA natural areas, vegetation was sampled and evaluated separately according to different habitats. As a result of the evaluation of 279 sampled areas, a total of 29 associations, 16 of which were new, were identified in three main habitat groups. These associations were represented by the Phragmito-Magnocaricetea, Potametea, Ammophiletea, Salicornietea fruticosae, Juncetea maritimi, and Molinio-Juncetea classes. In addition to these classes, Stellerietea media and Saginetea maritima classes are also represented as a result of the increase in species diversity and mono-recurrence species in the areas destroyed by grazing pressure and anthropogenic impacts. There are even places where sand dune vegetation is represented by the Quercetea ilicis class.

Some of the endemic and rare plants distributed in the research area could not be characteristic of any syntaxa, although they recur more than once in the synoptic table (Table 9). However, endemic *Onopordum boissieri* L. in the endangered category LR (nt), rare plants *Ambrosia maritima* L. in the endangered category EN and *Chlamydophora tridentata* (Delile) Ehrenb. Ex Less. in the endangered category VU were identified as the alliance characteristics of the habitat where they are distributed. Accordingly, *Onopordum boissieri* L. and *Ambrosia maritima* L. are added as new characteristic species for alliance *Euphorbio-Ammophilion arundinaceae* Br.-Bl. (1931) 1933 em. J.M. et J. Gehu 1988; *Chlamydophora tridentata* (Delile) Ehrenb. Ex Less. 1933 is added as a new characteristic species for the *Salicornion fruticosae* Br.-Bl. 1933 alliance.

In the research area, aquatic vegetation was represented by four associations belonging to the Phragmito-Magnocaricetea and Potametea classes. The subgroups of aquatic vegetation were also considered separately. Accordingly, in-channel vegetation was represented by two associations (one of which was new); in-lake vegetation was represented by one new association; and river and lake edge vegetation was represented by one association.

In the sand dune vegetation, the Ammophiletea Br.-Bl. & Tüxen ex Westhoff, Dijk, & Passchier 1946 class was identified with 12 associations. Among the subgroups identified in the sand dune vegetation, the most associations were found in the moving coastal sand dune vegetation (four associations—one new). Three new associations have been identified in fixed or semi-shifting sand dune vegetation, and three new associations in dune scrub vegetation. The damaged sand dune vegetation was represented by two new associations.

The halophytic vegetation was represented by 13 associations, eight of which belonged to Salicornietea fruticosae Br.-Bl. & Tüxen ex A. & O. Bolòs 1950, eight to Juncetea maritimi Br.-Bl. in Br.-Bl., Roussine & Nègre 1952, and one to Molinio-Juncetea Br.-Bl. (1931) 1947 classes. In the habitat, three associations (two of which are new) were identified in swamp vegetation. In the temporary pond subgroup, three associations were identified; in the temporary flooded flatland vegetation, five associations (two new) were identified. Halophytic terrestrial flatland vegetation was represented by two associations.

The alteration of the natural structure of the research area has been achieved through a variety of methods, including the draining of Tekfur marsh, the formulation of irrigation plans, and the allocation of drainage water to Akgöl-Paradeniz, among other actions. These modifications have resulted in a change in the water salinity of Akgöl. Nevertheless, it has been demonstrated that the region does indeed offer certain economic benefits in terms of agriculture and fisheries. Settlements, industrial facilities, pastures, and agricultural lands within the SEPA exert a direct or indirect influence on natural areas. Consequently, water, soil, and air are contaminated. The occurrence of both large and small accidental fires has been documented during the process of agricultural land acquisition or stubble burning in the designated research area. In dune areas, activities such as sand extraction, unauthorised plant removal, and cutting are carried out. Given the higher distribution of endemic and rare plant species in these habitats compared to other regions, it is imperative to enhance the frequency of inspections. In order to protect natural areas, ecotourism (e.g., bird watching, botany, and bicycle tourism) can be directed towards different applications, such as ecotourism in these areas, with a view to enabling local people to sustain their lives and earn an income.

## 4. Conclusions

The vegetation of the Göksu Delta RAMSAR area was classified according to habitat differences. As a result of the evaluation of 279 sampled areas, a total of 29 associations, 16 of which were new, were identified in three main vegetations. In the research area, there are associations mostly belonging to Ammophiletea, then Salicornietea fruticosae, Juncetea maritimi, Phragmito-Magnocaricetea, and least to Potametea and Molinio-Juncetea classes. In addition to these classes, Stellerietea media and Saginetea maritima classes are also represented as a result of the increase in species diversity and monotypic species in places that have been destroyed as a result of grazing pressure and anthropogenic impacts in the area. There are also places where dune vegetation is represented by the Quercetea ilicis class.

The endemic *Onopordum boissieri* L. is in the LR (nt) endangered category, the rare plant *Ambrosia maritima* L. is in the EN endangered category, and *Chlamydophora tridentata* (Delile) Ehrenb. Ex Less. in the VU endangered category were identified as the characteristic of the alliances of the habitats in which they are distributed. Accordingly, *Onopordum boissieri* L. and *Ambrosia maritima* L., *Euphorbio-Ammophilion arundinaceae* Br.-Bl. (1931) 1933 em. J.M. et J. Gehu 1988 and *Chlamydophora tridentata* (Delile) Ehrenb. Ex Less. to the *Salicornion fruticosae* Br.-Bl. 1933 was added as a new characteristic species for the alliance.

In the research area, aquatic vegetation was represented by four associations belonging to Phragmito-Magnocaricetea and Potametea classes. In the sand dune vegetation, Ammophiletea Br.-Bl. & Tüxen ex Westhoff, Dijk, & Passchier 1946 were identified with 12 associations belonging to the class. In the saline vegetation, it was represented by 13 associations. Eight of them were linked to Salicornietea fruticosae Br.-Bl. & Tüxen ex A. & O. Bolòs 1950, eight belonged to Juncetea maritimi Br.-Bl. in Br.-Bl., Roussine & Nègre 1952, and one belonged to Molinio-Juncetea Br.-Bl. (1931) 1947 classes.

The Göksu Delta generally preserved its natural characteristics until the 1980s. Since the early 1980s, the rapid increase in the number of second homes and their progress towards natural areas has been one of the most critical problems in the area. The rapid destruction and pollution of wetlands, plants, and bird breeding areas due to intensive agricultural activities in the area is one of the most important threats to the area. After the declaration of the delta as a Special Environmental Protection Area, construction has stopped; however, the activities of gaining agricultural land with fires could not be prevented. Endemic and endangered plants, especially on the coastal dunes, are in danger of extinction due to sand carrying and plant uprooting activities. The dams built on the Göksu River, which flows through the delta, are the most serious threat to sediment flow to the delta. All these are factors that may endanger the habitat and vegetation structure in the area. It is a natural area with rare ecosystems that has many important protection statuses, such as a natural site, a sensitive zone, a wildlife protection area, as well as an SEPA protection status. This area is one of the most important wetlands in Europe and shelters 12 of the 24 bird species in danger of extinction worldwide. It is also one of the 17 sea turtle breeding areas in Turkey. Twenty-nine plant associations have been identified in the area, 16 of which are new to the scientific world. Three of the endemic and rare plants have been added to the literature as alliance characteristics. In order to ensure the protection and sustainability of these associations, it is very important to monitor biodiversity in wetland ecosystems by controlling all threats in the area and regulating certain rules in terms of habitat management or conservation policy. This study is important in contributing to environmental protection and land use planning studies.

With this study, the vegetation of the area was studied in great detail, and syntaxonomic units found in different habitats were identified. The identified associations are given in Table A1 according to their main habitats and subgroups.

## Figures and Tables

**Table 1:** Similarity ratios of soil samples according to habitats by the fuzzy means 3 clustering method.

Habitat Types		Association Name	Clusters		
			1	2	3
Halophytic	Swamp	*Limonio gmelinii-Aeluropetum littoralis* (Bab. 1979) Gehu & Uslu 1989	0.49	0.28	0.23
		*Limonio angustifolii-Halimionetum portulacoides* ass. nov.	0.86	0.10	0.04
		*Asterisco aquaticae-Plantaginetum coronopi ssp. commutati* ass. nov.	0.23	0.05	0.71
	Temporary ponds	*Polypogono maritimi ssp. maritimi-Juncetum littoralis* Çakan, Düzenli, & Karaömerlioğlu 2003	0.02	0.01	0.98
		*Phragmiti australis-Juncetum maritimi* Vural, Duman, & al. 1995	0.07	0.90	0.02
		*Atriplici hastatae-Juncetum acuti* Çakan, Düzenli, & Karaömerlioğlu 2003	0.63	0.10	0.27
	Temporary flood plain	*Tamaricetum smyrensis* Seçmen & Leblebici 1996	0.80	0.14	0.06
		*Arthrocnemo fruticosii-Tamaricetum tetragynae* ass. nov.	0.92	0.05	0.03
		*Salicornio fragilis-Tamaricetum tetrandrae* ass. nov.	0.84	0.08	0.07
		*Schoeno nigricantis-Saccharetum ravennae* Çakan, Düzenli, & Karaömerlioğlu 2003	0.01	0.00	0.99
		*Eriantho-Schoenotum nigricantis* (Pign. 1953) Gehu 1984	0.02	0.01	0.97
	Terrestrial flatness	*Salicornio europaeae-Arthrocnemetum fruticosum* Çakan, Düzenli, & Karaömerlioğlu 2003	0.74	0.13	0.13
		*Arthrocnemo-Halocnemetum strobilaceii* Oberd 1952	0.93	0.05	0.03
Aquatic	The river and lake edges	*Bolboschoeno maritimi var. cymos-Phragmitetum australis* Borhidi & Balogh 1970	0.11	0.85	0.04
Sand Dune	Moving coastal sand dune	*Cypero mucronati-Agropyretum juncei* Kühnholtz ex Br.-Bl. 1933	0.03	0.01	0.96
		*Cakilo maritimae-Zygophylletum albi* Çakan, Düzenli, & Karaömerlioğlu 2003	0.22	0.07	0.72
		*Eryngio maritimi-Pancratietum maritimi* Çakan, Düzenli, & Karaömerlioğlu 2003	0.25	0.07	0.68
		*Ambrosio maritimae-Pancratietum maritimi* ass. nov.	0.25	0.07	0.68
	Fixed or semi-shifting sand dune	*Parapholido incurvae-Thymelaeetum hirsutae* ass. nov.	0.10	0.02	0.87
		*Cypero capitati-Trachomitetum veneti ssp. sarmatiense* ass. nov.	0.11	0.04	0.85
		*Echio angustifolii-Ononidetum natrix ssp. hispanicae* ass. nov.	0.03	0.01	0.95
	Dune scrub	*Sorgho halepense var. halepense-Myrtetum communis ssp. communis* ass. nov.	0.12	0.05	0.83
		*Polygono equisetiformis-Viticetum agni-casti* ass. nov.	0.28	0.07	0.66
		*Ephedro campylopodae-Populetum euphraticae* ass. nov.	0.08	0.03	0.90
	Damaged sand dune	*Urgino maritimi-Asphodeletum aestivi* ass. nov.	0.05	0.01	0.94
		*Verbasco sinuati-Sarcopoterietum spinosi* ass. nov.	0.07	0.02	0.92

**Table 2:** Similarity ratios of water samples according to aquatic habitats by the fuzzy means 3 clustering method.

Aquatic Habitat Types	Association Name	Winter Clusters			Spring Clusters			Summer Clusters			Autumn Clusters		
		1	2	3	1	2	3	1	2	3	1	2	3
In the channel	*Ludwigio stoloniferae-Nasturtietum officinalis* ass. nov.	0.00	0.00	1.00	0.00	0.00	1.00	0.00	0.00	1.00	0.00	0.00	1.00
		0.00	0.00	1.00	0.00	0.00	1.00	0.00	0.00	1.00	0.00	0.00	1.00
	*Potamogetono pectinati-Ceratophylletum demersi* (Hild & Rehnelt 1965) Passarge 1995	0.00	0.00	1.00	0.00	0.00	1.00	0.00	0.00	1.00	0.00	0.00	1.00
		0.00	0.00	1.00	0.00	0.00	1.00	0.00	0.00	1.00	0.00	0.00	1.00
In the lake	*Ruppio cirrhosae-Schoenoplectetum litoralis* ass. nov.	0.97	0.01	0.02	0.00	0.01	0.99	0.00	0.02	0.98	0.02	0.00	0.97
		0.37	0.03	0.60	0.00	0.02	0.98	0.00	0.10	0.89	0.99	0.01	0.01
The river and lake edges	*Bolboschoeno maritimi var. cymos-Phragmitetum australis* Borhidi & Balogh 1970	0.00	0.00	1.00	0.00	0.00	1.00	0.00	0.02	0.98	0.00	0.00	1.00
		0.97	0.01	0.02	0.00	0.01	0.99	0.00	0.02	0.98	0.02	0.00	0.97

**Table 3:** Aquatic vegetation types, associations, sample area number, sample area size, altitude, sampling time, and GPS coordinates.

Vegetation Types		Associations	Sample Area Number	Sample Area Size	Altitude	Sampling Time	GPS Coordinates	Sample Area Number	Sample Area Size	Altitude	Sampling Time	GPS Coordinates
Aquatic Vegetation	The vegetation in the channel	*Potamogetono pectinati-Ceratophylletum demersi* (Hild & Rehnelt 1965) Passarge 1995	361		0 m	24.07.2004	N: 36°19′931″	363		0 m	24.07.2004	N: 36°20′292″
				25 m^2^			E: 33°56′224″		50 m^2^			E: 34°56′159″
			347		0 m	23.07.2004	N: 36°20′336″	652		0 m	17.05.2005	N: 36°20′355″
				25 m^2^			E: 34°04′558″		50 m^2^			E: 33°58′103″
			274		0 m	20.07.2004	N: 36°23′654″	348		0 m	23.07.2004	N: 36°20′338″
				50 m^2^			E: 34°03′163″		50 m^2^			E: 34°04′560″
		*Ludwigio stoloniferae-Nasturtietum officinalis* ass. nov.	270		0 m	20.07.2004	N: 36°23′660″	278		0 m	20.07.2004	N: 36°23′836″
				50 m^2^			E: 34°03′196″		100 m^2^			E: 34°03′967″
			271		0 m	20.07.2004	N: 36°23′650″	317		0 m	22.07.2004	N: 36°22′600″
				50 m^2^			E: 34°03′142″		100 m^2^			E: 34°02′577″
			273		0 m	20.07.2004	N: 36°23′658″	279		0 m	20.07.2004	N: 36°23′838″
				50 m^2^			E: 34°03′204″		100 m^2^			E: 34°04′010″
	The vegetation in the lake	*Ruppio cirrhosae-Schoenoplectetum litoralis* ass. nov.	584		0 m	15.05.2005	N: 36°18′641″	686		0 m	19.05.2005	N: 36°17′970″
				25 m^2^			E: 34°03′690″		25 m^2^			E: 33°56′252″
			623		0 m	16.05.2005	N: 36°18′655″	659		0 m	18.05.2005	N: 36°17′980″
				25 m^2^			E: 33°58′447″		100 m^2^			E: 34°02′688″
			531		0 m	13.05.2005	N: 36°19′490″	688		0 m	19.05.2005	N: 36°17′315″
				50 m^2^			E: 34°04′398″		100 m^2^			E: 33°56′876″
			678		0 m	19.05.2005	N: 36°17′904″	523		0 m	13.05.2005	N: 36°19′810″
				25 m^2^			E: 33°57′928″		100 m^2^			E: 34°04′535″
			680		0 m	19.05.2005	N: 36°17′903″					
				25 m^2^			E: 33°58′447″					
	Vegetation of the river and lake edges	*Bolboschoeno maritimi* var. *cymos-Phragmitetum australis* Borhidi & Balogh 1970	649		0 m	17.05.2005	N: 36°20′067″	685		0 m	19.05.2005	N: 36°18′440″
				36 m^2^			E: 33°57′051″		36 m^2^			E: 33°56′902″
			681		0 m	19.05.2005	N: 36°17′958″	679		0 m	19.05.2005	N: 36°17′912″
				36 m^2^			E: 33°58′605″		36 m^2^			E: 33°58′152″
			372		0 m	24.07.2004	N: 36°16′972″	684		0 m	19.05.2005	N: 36°18′206″
				36 m^2^			E: 33°57′165″		36 m^2^			E: 33°57′738″
			529		10 cm	13.05.2005	N: 36°19′541″	578		0 m	15.05.2005	N: 36°18′558″
				36 m^2^			E: 34°04′477″		64 m^2^			E: 34°03′635″
			57		30 m	10.06.2004	N: 36°17′855″	657		0 m	18.05.2005	N: 36°18′099″
				36 m^2^			E: 33°55′664″		64 m^2^			E: 34°02′717″
			648		0 m	17.05.2005	N: 36°19′106″	539		10 cm	13.05.2005	N: 36°19′333″
				36 m^2^			E: 33°56′804″		36 m^2^			E: 34°04′369″
			64		0 m	11.06.2004	N: 36°17′822″	607		0 m	16.05.2005	N: 36°18′868″
				36 m^2^			E: 33°55′652″		64 m^2^			E: 33°57′263″
			350		0 m	23.07.2004	N: 36°20′300″	677		0 m	19.05.2005	N: 36°17′400″
				36 m^2^			E: 34°04′539″		36 m^2^			E: 33°58′020″
			344		0 m	23.07.2004	N: 36°20′272″	40		0 m	22.05.2004	N: 36°17′906″
				64 m^2^			E: 34°04′455″		36 m^2^			E: 33°55′621″
			609		0 m	16.05.2005	N: 36°18′860″					
				36 m^2^			E: 33°57′407″					

**Table 4:** Sand dunes vegetation types I, associations, sample area number, sample area size, altitude, sampling time and GPS coordinates.

Vegetation Types		Associations	Sample Area Number	Sample Area Size	Altitude	Sampling Time	GPS Coordinates	Sample Area Number	Sample Area Size	Altitude	Sampling Time	GPS Coordinates
Sand dune vegetation	Moving coastal sand dune vegetation	*Cakilo maritimae-Zygophylletum albi* Çakan, Düzenli & *Cakilo maritimae-Zygophylletum albi* Çakan, Düzenli, & Karaömerlioğlu 2003	180		0 m	14.07.2004	N: 36°17′354″	168		0 m	13.07.2004	N: 36°17′243″
				16 m^2^			E: 33°56′189″		16 m^2^			E: 33°56′235″
			166		0 m	13.07.2004	N: 36°17′068″	507		40–50 cm	29.04.2005	N: 36°18′094″
				16 m^2^			E: 33°56′507″		16 m^2^			E: 34°01′351″
		*Cypero mucronati-Agropyretum juncei* Kühnholtz ex Br.-Bl. 1933	188		0–20 cm	15.07.2004	N: 36°16′689″	194		0 m	15.07.2004	N: 36°16′282″
				25 m^2^			E: 33°56′669″		25 m^2^			E: 33°57′340″
			221		30 cm	16.07.2004	N: 36°15′841″	219		30 cm	16.07.2004	N: 36°15′770″
				25 m^2^			E: 33°58′277″		25 m^2^			E: 33°58′424″
			218		60 cm	16.07.2004	N: 36°15′772″	223		20 cm	16.07.2004	N: 36°15′787″
				25 m^2^			E: 33°58′429″		25 m^2^			E: 33°58′481″
			198		20 cm	15.07.2004	N: 36°16′504″	190		30 cm	15.07.2004	N: 36°16′459″
				25 m^2^			E: 33°57′255″		25 m^2^			E: 33°56′855″
			222		20 cm	16.07.2004	N: 36°15′790″	189		0–20 cm	15.07.2004	N: 36°16′643″
				25 m^2^			E: 33°58′454″		25 m^2^			E: 33°56′695″
			220		50 cm	16.07.2004	N: 36°15′827″	191		50 cm	15.07.2004	N: 36°16′432″
				25 m^2^			E: 33°58′352″		25 m^2^			E: 33°56′864″
		*Eryngio maritimi-Pancratietum maritimi* Çakan, Düzenli, & Karaömerlioğlu 2003	303		50 cm	21.07.2004	N: 36°21′714″	299		30 cm	21.07.2004	N: 36°21′557″
				25 m^2^			E: 34°04′663″		25 m^2^			E: 34°04′674″
			184		20 cm	14.07.2004	N: 36°16′952″	302		30 cm	21.07.2004	N: 36°21′691″
				25 m^2^			E: 33°56′532″		25 m^2^			E: 34°04′666″
			563		70 cm	14.05.2005	N: 36°18′119″	187		0–20 cm	15.07.2004	N: 36°16′700″
				25 m^2^			E: 34°03′187″		25 m^2^			E: 33°56′708″
			300		30 cm	21.07.2004	N: 36°21′575″	565		30 cm	14.05.2005	N: 36°18′195″
				25 m^2^			E: 34°04′679″		25 m^2^			E: 34°03′252″
		*Ambrosio maritimae-Pancratietum maritimi* ass. nov.	505		1 m	29.04.2005	N: 36°18′115″	570		60 cm	14.05.2005	N: 36°18′362″
				25 m^2^			E: 34°01′315″		25 m^2^			E: 34°03′291″
			510		1 m	29.04.2005	N: 36°18′140″	514		60 cm	29.04.2005	N: 36°18′195″
				25 m^2^			E: 34°01′550″		25 m^2^			E: 34°01′645″
			577		30 cm	15.05.2005	N: 36°18′523″	484		80 cm	28.04.2005	N: 36°18′099″
				25 m^2^			E: 34°03′638″		25 m^2^			E: 34°01′254″
			516		1 m	29.04.2005	N: 36°18′152″					
				25 m^2^			E: 34°01′679″					
	Fixed or semi-shifting sand dune vegetation	*Cypero capitati-Trachomitetum veneti* ssp. *sarmatiense* ass. nov.	562		20 cm	14.05.2005	N: 36°18′149″	286		0 m	20.07.2004	N: 36°22′334″
				25 m^2^			E: 34°03′135″		25 m^2^			E: 34°04′638″
			12		0 m	20.05.2004	N: 36°18′049″	306		1 m	21.07.2004	N: 36°21′843″
				16 m^2^			E: 33°55′399″		16 m^2^			E: 34°04′640″
			307		50 cm	21.07.2004	N: 36°21′986″	287		0 m	20.07.2004	N: 36°22′369″
				25 m^2^			E: 34°04′636″		16 m^2^			E: 34°04′634″
			304		80 cm	21.07.2004	N: 36°21′792″	305		1 m	21.07.2004	N: 36°21′836″
				16 m^2^			E: 34°04′652″		16 m^2^			E: 34°04′645″
		*Echio angustifolii-Ononidetum natrix* ssp. *hispanicae* ass. nov.	481		1 m	28.04.2005	N: 36°18′096″	386		50 cm	23.04.2005	N: 36°16′895″
				16 m^2^			E: 34°01′228″		25 m^2^			E: 33°57′288″
			441		0 m	25.04.2005	N: 36°17′059″	385		50 cm	23/4/2005/	N: 36°16′959″
				16 m^2^			E: 33°58′735″		16 m^2^			E: 33°57′251″
			24		80 cm	21.05.2004	N: 36°18′067″	208		60 cm	15.07.2004	N: 36°16′629″
				25 m^2^			E: 33°53′493″		16 m^2^			E: 33°57′211″
			211		60 cm	15.07.2004	N: 36°16′488″	308		1 m	21.07.2004	N: 36°22′017″
				16 m^2^			E: 33°57′580″		16 m^2^			E: 34°04′579″
			558		40 cm	14.05.2005	N: 36°18′141″	312		1 m	21.07.2004	N: 36°21′959″
				16 m^2^			E: 34°03′092″		16 m^2^			E: 34°04′450″
			282		1.5 m	20.07.2004	N: 36°22′331″	284		2 m	20.07.2004	N: 36°22′319″
				16 m^2^			E: 34°04′467″		16 m^2^			E: 34°04′532″
			203		70 cm	15.07.2004	N: 36°16′683″					
				16 m^2^			E: 33°57′206″					
		*Parapholido incurvae-Thymelaeetum hirsutae* ass. nov.	405		40 cm	24.04.2005	N: 36°16′919″	165		30 cm	13.07.2004	N: 36°16′900″
				16 m^2^			E: 33°58′544″		16 m^2^			E: 33°56′657″
			404		40 cm	24.04.2005	N: 36°16′911″	167		80 cm	13.07.2004	N: 36°17′228″
				16 m^2^			E: 33°58′534″		25 m^2^			E: 33°56′318″
			482		70 cm	28.04.2005	N: 36°18′096″	181		30 cm	14.07.2004	N: 36°17′100″
				25 m^2^			E: 34°01′213″		25 m^2^			E: 33°56′097″
			107		0 m	15.06.2004	N: 36°17′490″					
				16 m^2^			E: 33°56′048″					

**Table 5:** Sand dune vegetation type II: associations, sample area number, sample area size, altitude, sampling time and GPS coordinates.

Vegetation Types		Associations	Sample Area Number	Sample Area Size	Altitude	Sampling Time	GPS Coordinates	Sample Area Number	Sample Area Size	Altitude	Sampling Time	GPS Coordinates
Sand dune vegetation	Dune scrub vegetation	*Sorgho halepense* var. *halepense-Myrtetum communis* ssp.	69		50 cm	11.06.2004	N: 36°17′737″	87		2 m	13.06.2004	N: 36°17′622″
				36 m^2^			E: 33°56′037″		36 m^2^			E: 33°55′966″
			148		50 cm	12.07.2004	N: 36°17′052″	44		3 m	22.05.2004	N: 36°17′879″
				36 m^2^			E: 33°56′782″		36 m^2^			E: 33°55′655″
			70		0 m	11.06.2004	N: 36°17′750″	98		80 cm	13.06.2004	N: 36°17′612″
				16 m^2^			E: 33°56′017″		16 m^2^			E: 33°56′000″
			113		1 m	15.06.2004	N: 36°17′575″					
				16 m^2^			E: 33°56′202″					
		*Polygono equisetiformis-Viticetum agni-casti* ass. nov. birliği	48		0 m	29.05.2004	N: 36°17′893″	43		1.5 m	22.05.2004	N: 36°17′880″
				16 m^2^			E: 33°55′518″		16 m^2^			E: 33°55′648″
			68		0 m	11.06.2004	N: 36°17′805″	201		8 m	15.07.2004	N: 36°16′658″
				25 m^2^			E: 33°55′678″		25 m^2^			E: 33°57′215″
			147		1 m	12.07.2004	N: 36°17′088″					
				25 m^2^			E: 33°56′763″					
		*Ephedro campylopodae-Populetum euphraticae* ass. nov.	92		1 m	13.06.2004	N: 36°17′684″	309		2 m	21.07.2004	N: 36°21′996″
				50 m^2^			E: 33°56′047″		50 m^2^			E: 34°04′550″
			136		30 cm	16.06.2004	N: 36°17′350″	163		50 cm	13.07.2004	N: 36°16′828″
				25 m^2^			E: 33°56′375″		25 m^2^			E: 33°57′125″
			91		1 m	13.06.2004	N: 36°17′703″	311		7.5 m	21.07.2004	N: 36°21′978″
				25 m^2^			E: 33°56′015″		50 m^2^			E: 34°04′482″
			140		3 m	17.06.2004	N: 36°17′321″	310		8 m	21.07.2004	N: 36°21′980″
				50 m^2^			E: 33°56′568″		50 m^2^			E: 34°04′520″
			313		1.5 m	21.07.2004	N: 36°21′939″					
				50 m^2^			E: 34°04′525″					
	Damaged sand dune vegetation	*Urgino maritimi-Asphodeletum* aestivi ass. nov.	406		10 cm	24.04.2005	N: 36°16′913″	402		30 cm	24.04.2005	N: 36°16′906″
				25 m^2^			E: 33°58′494″		16 m^2^			E: 33°58′492″
			401		30 cm	24.04.2005	N: 36°16′904″					
				16 m^2^			E: 33°58′498″					
		*Verbasco sinuati-Sarcopoterietum spinosi* ass. nov.	486		50 cm	28.04.2005	N: 36°18′135″	75		80 cm	12.06.2004	N: 36°17′824″
				25 m^2^			E: 34°01′287″		16 m^2^			E: 33°55′720″
			106		80 cm	15.06.2004	N: 36°17′544″	54		0 m	20.05.2004	N: 36°17′860″
				16 m^2^			E: 33°56′077″		16 m^2^			E: 33°55′613″
			38		1 m	22.05.2004	N: 36°17′974″	50		50 cm	29.05.2004	N: 36°17′900″
				16 m^2^			E: 33°55′580″		16 m^2^			E: 33°55′536″
			197		0 m	15.07.2004	N: 36°16′481″	96		1 m	13.06.2004	N: 36°17′629″
				25 m^2^			E: 33°57′430″		16 m^2^			E: 33°56′014″
			84		0 m	13.06.2004	N: 36°17′683″	123		1 m	16.06.2004	N: 36°17′515″
				25 m^2^			E: 33°55′862″		16 m^2^			E: 33°56′200″

**Table 6:** Halophytic vegetation types I: associations, sample area number, sample area size, altitude, sampling time, and GPS coordinates.

Vegetation Types		Associations	Sample Area Number	Sample Area Size	Altitude	Sampling Time	GPS Coordinates	Sample Area Number	Sample Area Size	Altitude	Sampling Time	GPS Coordinates
Halophytic vegetation	Halophytic vegetation of the swamp	*Limonio gmelinii-Aeluropetum littoralis* (Bab. 1979) Gehu & Uslu 1989	102		0 m	14.06.2004	N: 36°17′625″	146		0 m	17.06.2004	N: 36°17′246″
				9 m^2^			E: 33°56′132″		9 m^2^			E: 33°56′529″
			121		0 m	16.06.2004	N: 36°17′540″	145		0 m	17.06.2004	N: 36°17′209″
				9 m^2^			E: 33°56′277″		9 m^2^			E: 33°56′564″
		*Limonio angustifolii-Halimionetum portulacoides* ass. nov.	446		10 cm	26.04.2005	N: 36°17′135″	423		5 cm	25.04.2005	N: 36°16′994″
				25 m^2^			E: 33°57′786″		25 m^2^			E: 33°58′482″
			246		0 m	17.07.2004	N: 36°17′101″	501		0 m	28.04.2005	N: 36°18′663″
				16 m^2^			E: 33°57′563″		16 m^2^			E: 34°01′698″
			411		10 cm	24.04.2005	N: 36°16′920″	502		0 m	28.04.2005	N: 36°18′673″
				16 m^2^			E: 33°58′523″		25 m^2^			E: 34°01′727″
			250		0 m	17.07.2004	N: 36°17′159″	503		0 m	28.04.2005	N: 36°18′675″
				25 m^2^			E: 33°57′817″		16 m^2^			E: 34°01′735″
			444		10 m	25.04.2005	N: 36°17′107″	254		0 m	19.07.2004	N: 36°16′643″
				25 m^2^			E: 33°58′696″		16 m^2^			E: 33°58′477″
			447		10 cm	26.04.2005	N: 36°17′147″	255		0 m	19.07.2004	N: 36°18′683″
				16 m^2^			E: 33°57′810″		25 m^2^			E: 33°58′476″
			267		0 m	19.07.2004	N: 36°17′757″	438		0 m	25.04.2005	N: 36°17′050″
				16 m^2^			E: 33°58′725″		25 m^2^			E: 33°58′670″
			259		0 m	19.07.2004	N: 36°16′975″	696		20 cm	20.05.2005	N: 36°18′483″
				16 m^2^			E: 33°59′467″		25 m^2^			E: 34°02′513″
			420		0 m	24.04.2005	N: 36°16′957″	695		20 cm	20.05.2005	N: 36°18′452″
				25 m^2^			E: 33°58′465″		25 m^2^			E: 34°02′559″
		*Asterisco aquaticae-Plantaginetum coronopi* ssp. *commutati* ass. nov.	384		40 cm	23.04.2005	N: 36°16′961″	414		10 cm.	24.04.2005	N: 36°16′941″
				16 m^2^			E: 33°57′227″		16 m^2^			E: 33°58′558″
			450		10 cm	26.04.2005	N: 36°17′211″	382		60 cm	23.04.2005	N: 36°16′934″
				25 m^2^			E: 33°57′966″		16 m^2^			E: 33°57′236″
			399		40 cm	23.04.2005	N: 36°16′995″	421		10 cm	25.04.2005	N: 36°17′017″
				16 m^2^			E: 33°57′375″		16 m^2^			E: 33°58′469″
			425		10 cm	25.04.2005	N: 36°17′023″	398		40 cm	23.04.2005	N: 36°17′034″
				25 m^2^			E: 33°58′506″		16 m^2^			E: 33°57′412″
			426		10 cm	25.04.2005	N: 36°17′014″	383		40 cm	23.04.2005	N: 36°16′949″
				16 m^2^			E: 33°58′486″		16 m^2^			E: 33°57′231″
			430		10 cm	25.04.2005	N: 36°17′053″	437		10 cm	25.04.2005	N: 36°17′048″
				25 m^2^			E: 33°58′565″		25 m^2^			E: 33°58′658″
			385		50 cm	23.04.2005	N: 36°16′959″					
				16 m^2^			E: 33°57′251″					
	Halophytic vegetation of temporary ponds	*Polypogono maritimi* ssp. *maritimi-Juncetum littoralis* Çakan, Düzenli, & Karaömerlioğlu 2003	662		10 cm	18.05.2004	N: 36°18′008″	150		0 m	12.07.2004	N: 36°17′096″
				25 m^2^			E: 34°02′773″		16 m^2^			E: 33°56′671″
			132		0 m	16.06.2004	N: 36°17′404″	10		0 m	20.05.2004	N: 36°18′021″
				16 m^2^			E: 33°56′336″		16 m^2^			E: 33°55′375″
			144		0 m	17.06.2004	N: 36°17′223″	515		0 m	29.04.2005	N: 36°18′205″
				16 m^2^			E: 33°56′566″		16 m^2^			E: 34°01′664″
			288		0 m	20.07.2004	N: 36°22′378″	108		0 m	15.06.2004	N: 36°17′470″
				25 m^2^			E: 34°04′581″		16 m^2^			E: 33°55′071″
			470		10 cm	27.04.2005	N: 36°18′800″	47		0 m	22.05.2004	N: 36°17′923″
				16 m^2^			E: 34°00′469″		16 m^2^			E: 33°55′555″
		*Phragmiti australis-Juncetum maritimi* Vural, Duman, & al. 1995	557		0 m	14.05.2005	N: 36°18′097″	569		0 m	14.05.2005	N: 36°18′348″
				16 m^2^			E: 34°03′077″		36 m^2^			E: 34°03′235″
			596		0 m	15.05.2005	N: 36°19′185″	527		10 cm	13.05.2005	N: 36°19′873″
				36 m^2^			E: 34°04′347″		16 m^2^			E: 34°04′447″
			663		0 m	18.05.2005	N: 36°17′900″	622		0 m	16.05.2005	N: 36°18′695″
				25 m^2^			E: 34°02′750″		36 m^2^			E: 33°58′451″
			588		0 m	15.05.2005	N: 36°18′732″	580		0 m	15.05.2005	N: 36°18′571″
				25 m^2^			E: 34°03′926″		36 m^2^			E: 34°03′673″
			550		10 cm	14.05.2005	N: 36°17′974″	597		0 m	15.05.2005	N: 36°19′175″
				16 m^2^			E: 34°02′980″		16 m^2^			E: 34°04′176″
		*Atriplici hastatae-Juncetum acuti* Çakan, Düzenli, & Karaömerlioğlu 2003	603		0 m	16.05.2005	N: 36°19′096″	469		5 cm	27.04.2005	N: 36°18′803″
				25 m^2^			E: 33°57′336″		25 m^2^			E: 34°00′435″
			618		0 m	16.05.2005	N: 36°19′049″	534		10 cm	13.05.2005	N: 36°19′755″
				16 m^2^			E: 33°58′053″		16 m^2^			E: 34°04′253″
			504		0 m	28.04.2005	N: 36°19′894″	468		10 cm	27.04.2005	N: 36°18′822″
				25 m^2^			E: 34°00′280″		25 m^2^			E: 34°00′413″
			626		0 m	16.05.2005	N: 36°21′236″	615		0 m	16.05.2005	N: 36°18′972″
				36 m^2^			E: 34°03′703″		36 m^2^			E: 33°57′890″
			487		0 m	28.04.2005	N: 36°18′117″	691		50 cm	20.05.2005	N: 36°18′590″
				36 m^2^			E: 34°01′236″		16 m^2^			E: 34°02′566″

**Table 7:** Halophytic vegetation type II: associations, sample area number, sample area size, altitude, sampling time, and GPS coordinates.

Vegetation Types		Associations	Sample Area Number	Sample Area Size	Altitude	Sampling Time	GPS Coordinates	Sample Area Number	Sample Area Size	Altitude	Sampling Time	GPS Coordinates
Halophytic vegetation	Halophytic vegetation of temporary floodplain	*Tamaricetum smyrensis* Seçmen & Leblebici 1996	676		0 m	18.05.2005	N: 36°18′208″	538		10 cm	13.05.2005	N: 36°19′355″
				25 m^2^			E: 34°02′346″		25 m^2^			E: 34°04′305″
			525		45 cm	13.05.2005	N: 36°19′810″	544		60 cm	13.05.2005	N: 36°19′606″
				16 m^2^			E: 34°04′525″		16 m^2^			E: 34°04′566″
			520		10 cm	13.05.2005	N: 36°19′666″	705		0 m	20.05.2005	N: 36°19′050″
				25 m^2^			E: 34°04′384″		25 m^2^			E: 34°02′279″
		*Arthrocnemo fruticosii-Tamaricetum tetragynae* ass. nov.	25		0 m	21.05.2004	N: 36°18′108″	340		0 m	23.07.2004	N: 36°20′345″
				36 m^2^			E: 33°55′513″		64 m^2^			E: 34°04′386″
			633		20 cm	17.05.2005	N: 36°18′121″	541		40 cm	13.05.2005	N: 36°19′471″
				64 m^2^			E: 33°57′646″		64 m^2^			E: 34°04′525″
			354		50 cm	23.07.2004	N: 36°20′371″	646		0 m	17.05.2005	N: 36°18′471″
				64 m^2^			E: 34°04′569″		36 m^2^			E: 33°57′461″
			298		0 m	21.07.2004	N: 36°21′844″	264		0 m	19.07.2004	N: 36°17′435″
				36 m^2^			E: 34°04′323″		36 m^2^			E: 33°58′505″
			364		0 m	24.07.2004	N: 36°20′292″	328		20 cm	22.07.2004	N: 36°20′287″
				36 m^2^			E: 33°56′159″		64 m^2^			E: 34°04′719″
			555		0 m	14.05.2005	N: 36°18′066″	330		0 m	22.07.2004	N: 36°20′220″
				36 m^2^			E: 34°03′120″		64 m^2^			E: 34°04′651″
			266		0 m	19.07.2004	N: 36°17′644″	329		1 m	22.07.2004	N: 36°20′218″
				36 m^2^			E: 33°58′483″		64 m^2^			E: 34°04′712″
			631		0 m	17.05.2005	N: 36°18′135″	243		0 m	17.07.2004	N: 36°16′933″
				64 m^2^			E: 33°57′742″		64 m^2^			E: 33°57′550″
		*Salicornio fragilis-Tamaricetum tetrandrae* ass. nov.	702		50 cm	20.05.2005	N: 36°18′841″	654		0 m	18.05.2005	N: 36°18′192″
				100 m^2^			E: 34°01′993″		100 m^2^			E: 34°02′677″
			608		30 cm	16.05.2005	N: 36°18′869″	655		0 m	18.05.2005	N: 36°18′131″
				50 m^2^			E: 33°57′268″		100 m^2^			E: 34°02′701″
			698		10 cm	20.05.2005	N: 36°18′567″	598		30 cm	15.05.2005	N: 36°19′242″
				100 m^2^			E: 34°01′499″		50 m^2^			E: 34°04′209″
			703		20 cm	20.05.2005	N: 36°18′872″	697		0 m	20.05.2005	N: 36°18′524″
				100 m^2^			E: 34°02′014″		100 m^2^			E: 34°02′541″
			341		0 m	23.07.2004	N: 36°20′318″	689		0 m	19.05.2005	N: 36°17′267″
				50 m^2^			E: 34°04′306″		100 m^2^			E: 33°57′866″
			353		30 cm	23.07.2004	N: 36°20′334″	690		30 cm	20.05.2005	N: 36°18′582″
				50 m^2^			E: 34°04′578″		50 m^2^			E: 34°02′552″
			66		0 m	11.06.2004	N: 36°17′807″	658		0 m	18.05.2005	N: 36°18′020″
				50 m^2^			E: 33°55′659″		100 m^2^			E: 34°02′672″
			154		30 cm	13.07.2004	N: 36°16′974″	155		0 m	13.07.2004	N: 36°17′051″
				50 m^2^			E: 33°56′656″		50 m^2^			E: 33°56′845″
			653		0 m	18.05.2005	N: 36°18′231″	629		0 m	17.05.2005	N: 36°18′063″
				50 m^2^			E: 34°02′691″		50 m^2^			E: 33°57′073″
		*Schoeno nigricantis-Saccharetum ravennae* Çakan, Düzenli, & Karaömerlioğlu 2023	209		0 m	15.07.2004	N: 36°16′467″	37		0 m	22.05.2004	N: 36°17′931″
				25 m^2^			E: 33°57′251″		25 m^2^			E: 33°55′550″
			314		0 m	21.07.2004	N: 36°21′909″	315		0 m	21.07.2004	N: 36°21′887″
				25 m^2^			E: 34°04′537″		25 m^2^			E: 34°04′507″
			159		0 m	13.07.2004	N: 36°16′937″	202		0 m	15.07.2004	N: 36°16′666″
				25 m^2^			E: 33°56′748″		25 m^2^			E: 33°57′213″
		*Eriantho-Schoenotum nigricantis* (Pign. 1953) Gehu 1984	110		30 cm	15.06.2004	N: 36°17′515″	45		1 m	22.05.2004	N: 36°17′852″
				16 m^2^			E: 33°56′114″		16 m^2^			E: 33°55′601″
			77		0 m	12.06.2004	N: 36°17′860″	36		0 m	22.05.2004	N: 36°17′921″
				25 m^2^			E: 33°55′744″		16 m^2^			E: 33°55′493″
			86		0 m	13.06.2004	N: 36°17′556″	30		0 m	21.05.2004	N: 36°17′979″
				25 m^2^			E: 33°55′942″		16 m^2^			E: 33°55′469″
			127		0 m	16.06.2004	N: 36°17′421″					
				16 m^2^			E: 33°56′153″					

**Table 8:** Halophytic vegetation type III, associations, sample area number, sample area size, altitude, sampling time, and GPS coordinates.

Vegetation Types		Associations	Sample Area Number	Sample Area Size	Altitude	Sampling Time	GPS Coordinates	Sample Area Number	Sample Area Size	Altitude	Sampling Time	GPS Coordinates
Halophytic vegetation	Halophytic vegetation of terrestrial flatness	*Salicornio europaeae-Arthrocnemetum fruticosum* Çakan, Düzenli, & Karaömerlioğlu 2023	241		0 m	17.07.2004	N: 36°17′041″	532		10 cm	13.05.2005	N: 36°19′463″
				16 m^2^			E: 33°57′516″		36 m^2^			E: 34°04′354″
			242		0 m	17.07.2004	N: 36°17′014″	638		0 m	17.05.2005	N: 36°18′323″
				16 m^2^			E: 33°57′485″		36 m^2^			E: 33°57′907″
			642		40 cm	17.05.2005	N: 36°18′591″	251		0 m	17.07.2004	N: 36°17′330″
				16 m^2^			E: 33°57′713″		16 m^2^			E: 33°58′168″
			474		0 m	27.04.2005	N: 36°19′094″	326		0 m	22.07.2004	N: 36°20′471″
				16 m^2^			E: 34°00′375″		16 m^2^			E: 34°04′670″
			476		30 cm	27.04.2005	N: 36°19′088″	324		0 m	22.07.2004	N: 36°20′681″
				36 m^2^			E: 34°00′403″		36 m^2^			E: 34°04′673″
			268		0 m	19.07.2004	N: 36°17′733″	256		0 m	19.07.2004	N: 36°16′653″
				16 m^2^			E: 33°58′803″		16 m^2^			E: 33°58′539″
			323		0 m	22.07.2004	N: 36°20′646″	496		10 cm	28.04.2005	N: 36°18′709″
				36 m^2^			E: 34°04′651″		16 m^2^			E: 34°01′113″
			252		0 m	17.07.2004	N: 36°17′335″	213		0 m	16.07.2004	N: 36°16′343″
				16 m^2^			E: 33°58′196″		16 m^2^			E: 33°57′782″
			245		0 m	17.07.2004	N: 36°16′995″	620		0 m	16.05.2005	N: 36°18′915″
				16 m^2^			E: 33°57′569″		16 m^2^			E: 33°58′250″
			214		20 cm	16.07.2004	N: 36°16′378″	673		0 m	18.05.2005	N: 36°18′032″
				16 m^2^			E: 33°57′798″		16 m^2^			E: 34°02′468″
			322		0 m	22.07.2004	N: 36°20′712″					
				16 m^2^			E: 34°04′718″					
		*Arthrocnemo-Halocnemetum strobilaceii* Oberd 1952	216		40 cm	16.07.2004	N: 36°16′416″	261		0 m	19.07.2004	N: 36°17′209″
				25 m^2^			E: 33°58′041″		16 m^2^			E: 33°58′680″
			248		0 m	17.07.2004	N: 36°17′056″	196		0 m	15.07.2004	N: 36°16′457″
				16 m^2^			E: 33°57′575″		16 m^2^			E: 33°57′424″
			498		0 m	28.04.2005	N: 36°18′563″	262		0 m	19.07.2004	N: 36°17′449″
				16 m^2^			E: 34°01′104″		16 m^2^			E: 33°58′632″
			239		20 cm	17.07.2004	N: 36°16′807″	260		0 m	19.07.2004	N: 36°17′243″
				16 m^2^			E: 33°57′421″		16 m^2^			E: 33°58′748″
			478		40 cm	27.04.2005	N: 36°19′131″	672		0 m	18.05.2005	N: 36°18′009″
				25 m^2^			E: 34°00′469″		16 m^2^			E: 34°02′463″
			229		0 m	16.04.2004	N: 36°16′479″					
				16 m^2^			E: 33°59′111″					

**Table 9:** Synoptic table of vegetation according to habitats.

	Sand Dunes Vegetation													Halophytic Vegetation													Aquatic Vegetation												
Number of Sample Area	4	12	8	7	8	13	7	5	9	4	7	5	9	6	3	10	4	18	13	8	3	10	10	10	6	16	18	6	7	8	21	11	5	6	6	9	19	9	5
Number of Species	9	19	21	41	43	61	77	18	77	35	83	65	50	68	63	92	9	72	73	34	45	56	24	56	28	63	64	40	44	55	45	37	38	11	20	7	30	26	29
Ammophiletea Br.-Bl. & Tüxen ex Westhoff, Dijk & Passchier 1946																																							
*Crepis foetida subsp. foetida*				V		I	III		II	III	I	II	I	I	V	II		II								I	I				I	I	II						
*Bromus rigidus*						II			II	II	III	III	II	II	II	I	I	I									I			II			I						
*Centaurium pulchellum*							I		II	III	I			I	II	I		II	I		II	I						III	III		I								
*Centaurium erythraea subsp. rumelicum*									I	III						I		I				I		I			I	IV	I		I								
*Xanthium strumarium subsp. cavanillesii*		I	III		IV	I			II	III			I									I						I											I
*Bromus psamophilus*					I				I		II	II		III		I						I					I		III										
*Blackstonia perfoliata subsp. perfoliata*					I				II		I					I									I				II			I							
*Salsola kali*			IV		II	I														I						I	I			I									
*Medicago littoralis var. littoralis*				II		I			I			I				I		I	I																				
*Salsola ruthenica*		I	II			I														II					I	I													
*Medicago marina*			II		II		I																																
*Euphorbia peplus var. peplus*									I		I				II																								
*Maresia nana*												II				I																							
*Eryngium creticum*															II			I																					
*Alhagi mannifera*												I							I																				
Euphorbio-Ammophiletalia arundinaceae Br.-Bl. (1931) 1933 em. J.M. et J. Gehu 1988																																							
*Vulpia fasciculata*				V		II	I	IV	II	III	II	II	I	I		II										I			I	II			I						
*Trifolium purpureum var. purpureum*						I	II		II				I		V			I	IV		V																	I	I
*Euphorbio-Ammophilion arundinaceae* Br.-Bl. (1931) 1933 em. J.M. et J. Gehu 1988																																							
*Silene kotschyi var. maritima*				III		II	I	II	I		III	II	II	I		III																							
*Onopordum boissieri*						I		I			I	I	I	I	I																								
*Medicago minima var. minima*																		I									I												
*Euphorbia peplis*		II			II																																		
*Cakilo maritimae-Zygophylletum albi* Çakan, Düzenli & Karaömerlioğlu 2003																																							
*Zygophyllum album*	V	V	II				I													II		I							I			I							
*Cakile maritima*		I																																					
*Cypero mucronati-Agropyretum juncei* Kühnholtz ex Br.-Bl. 1933																																							
*Eryngium maritimum*		III	IV		II		I													I																			
*Elymus farctus subsp. bessarabicus var. bessarabicus*		V	II				II																							I									
*Sporobolus virginicus*	IV	I	II	II	II		I		II	III	I		I	I		I				IV		I	II			I	I				I								
*Euphorbia paralias*	II	V	III		I				II																					I									
*Eryngio maritimi-Pancratietum maritimi* Çakan, Düzenli & Karaömerlioğlu 2003																																							
*Pancratium maritimum*	II	V	V	V	IV	I	II		II	II																		I											
*Ipomoea stolonifera*			IV		I	I	I																																
*Otanthus maritimus*		I	I																																				
*Ambrosio maritimae-Pancratietum maritimi ass. nov.*																																							
*Ambrosia maritima*				V	I	II	II		III	III	III		II	II		II				I		I						I	I	II									
*Cypero capitati-Trachomitetum veneti ssp. sarmatiense ass. nov.*																																							
*Trachomitum venetum subsp. sarmatiense*					V						I											I						II											
*Cyperus capitatus*			IV		IV	II				III		I	II																										
*Echio angustifolii-Ononidetum natrix ssp. hispanicae ass. nov.*																																							
*Ononis natrix subsp. hispanica*			I	V	I	V	IV	IV	III	III	II	III	V	II		II														I									
*Echium angustifolium*			II	I	I	V	I	I	II	II		II	IV	II		II														II									
*Parapholido incurvae-Thymelaeetum hirsutae ass. nov.*																																							
*Thymelaea hirsuta*		I					V									I												I											
*Parapholis incurva*				III	I		IV		II	II		I		I	IV	I		IV	IV	II	V	I		II	II	I	II			II	III	IV	IV						
*Bromus rubens*							III								V			I	III		IV					I	I												
*Helianthemum stipulatum* society																																							
*Helianthemum stipulatum*								V			III	I				II																							
*Imperata cylindrica var. cylindrica-Trisetaria loeflingiana* society																																							
*Imperata cylindrica var. cylindrica*			I	I		I			V	I		I		II		I													II										I
*Trisetaria loeflingiana*				II	I		I		II			I		I	II			I	II		II			II	I	I	I	II	III			II	I						
*Inula viscosa* society																																							
*Inula viscosa*					II	I	I		III	V	I	II	I	II								II						V	III	II							I		
*Sorgho halepense var. halepense-Myrtetum communis ssp. communis ass. nov.*																																							
*Myrtus communis subsp. communis*							I				V	I		III															I										
*Sorghum halepense var. halepense*											V	I		II										I					I					I	IV				I
*Polygono equisetiformis-Viticetum agni-casti ass. nov.*																																							
*Vitex agnus-castus*									I	III	I	V	III	II		II												I	I										
*Polygonum equisetiforme*		II		III	II	II	III		II	III	V	IV	III	IV	IV	III		I	II	II	V	I		II		I	I	III	II	II			II						
*Ephedro campylopodae-Populetum euphraticae ass. nov.*																																							
*Populus euphratica*													V																										
*Ephedra campylopoda*											III		II	II																									
*Nerium oleander-Polypogon maritimus var. maritimus* society																																							
*Polypogon maritimus subsp. maritimus*									II	IV	II	I	I	IV		I		I				II		III	I		II	IV	IV	I	I		I					II	II
*Nerium oleander*									I		II	III	III	V														II											
*Urgino maritimi-Asphodeletum aestivi ass. nov.*																																							
*Asphodelus aestivus*							II	II			V	II	II	III	V	IV		I	I			I							I										
*Urginea maritima*							III						I		V	I																							
*Plantago cretica*							II								V																								
*Verbasco sinuati-Sarcopoterietum spinosi ass. nov.*																																							
*Sarcopoterium spinosum*				II	I		III	I	II	I	IV	II	I	III		V												I	III										
*Verbascum sinuatum var. sinuatum*						I	II	I	I	II	III	III	III	III	IV	IV														II									
*Allium junceum subsp. tridentatum*																I																							
Salicornietea fruticosae Br.-Bl. & Tüxen ex A. & O. Bolòs 1950																																							
*Rostraria cristata var. cristata*				II	I	I	III		III	II	I	I			V	III		II	III	I	V	I	I	I		I	I	I	II	I	I	III							
*Suaeda prostrata subsp. prostrata*					II				I								II		I		II	I	I	I	I	III	II		I	II	I		I				I		I
*Spergularia salina*																			I			I		I						I	II	I							I
*Triglochin bulbosa subsp. barrelieri*																						II							I			I	I						
*Carex divisa*																										I													
*Petrosimonia brachiata*																		I																					
*Salicornion fruticosae* Br.-Bl. 1933																																							
*Chlamydophora tridentata*																		II	II		V						I				II	I							
*Halopeplis amplexicaulis*																															I	I							
*Limonio angustifolii-Halimionetum portulacoides ass. nov.*																																							
*Halimione portulacoides*							I											V	III	III	IV	I	I	V	IV	IV	IV			II	IV	III	V						
*Limonium angustifolium*							I										III	IV	II	II	V	II		II	I	II	II	I	I	III	II	I	II						
*Hordeum murinum subsp. murinum*						I		II					II	I		I		V	I	I	IV			IV		II		I	II	II	II	III	III						
*Asterisco aquaticae-Plantaginetum coronopi ssp. commutati ass. nov.*																																							
*Plantago coronopus subsp. commutata*									II			I		I	IV			IV	V	I	V	I		II	I	I	I	I		I	II	IV	II						
*Asteriscus aquaticus*							II								V				IV		V																		
*Gynandriris sisyrinchium*															I	I		II	V		V					I													
*Inula crithmoides-Limonium graecum var. graecum society*																																							
*Limonium graecum var. graecum*	IV	I					III					I				I				II								II	I				I						
*Inula crithmoides*	IV	I	I				I		I											V		II	IV	II	IV	II	III			II	I	I							I
*Limonium virgatum-Carthamus tenuis ssp. tenuis society*																																							
*Limonium virgatum*		I		I					II									I	I	II	V		II				I		I			I							
*Carthamus tenuis subsp. tenuis*																					IV																		
*Salicornio europaeae-Arthrocnemetum fruticosum* Çakan, Düzenli & Karaömerlioğlu 2003																																							
*Arthrocnemum fruticosum*																	III	II		IV		I	IV		III	IV	I			II	V	IV	I				I		
*Halocnemion strobilacei* Gehu & Costa 1984																																							
*Tamaricetum smyrensis* Seçmen & Leblebici 1996																																							
*Tamarix smyrensis*																									V		I	I		I	I								
*Salsola soda*			I		II		II													I					I	I	I			I									
*Arthrocnemo-Halocnemetum strobilaceii* Oberd 1952																																							
*Halocnemum strobilaceum*																		I													II	V							
*Sphenopus divaricatus*																		II	I		IV	I					I				I	II							
*Arthrocnemion glauci* Rivas-Martínez & Costa 1984																																							
*Cakile maritima*	II		I				I													II						I													
*Elymus farctus subsp. bessarabicus var. striatulus*																						I				I						I	I						
*Cynodon dactylon var. villosus*																		I																					
*Arthrocnemum glaucum society*																																							
*Arthrocnemum glaucum*																		II	I			I	I		I	I	II				I	I	V						
Juncetea maritimi Br.-Bl. in Br.-Bl., Roussine & Nègre 1952																																							
*Spergularia bocconii*							II											I		I	II	I		II		I	I				I	I	I						
*Puccinellia distans subsp. distans*																						I		III															I
*Carex extensa*																						I	II																
*Elymus elongatus subsp. ponticus*																						I								I									
Juncetalia maritimi Br.-Bl. 1931 em. Julve 1992 ex 1993																																							
*Juncion maritimi* Br.-Bl. 1931 em. Julve 1992 ex 1993																																							
*Melilotus alba*					I				II	I	I							II	I	I	IV			II		I	I					I	I						
*Saccharum ravennae*					I				II	I	I							II	I	I	IV			II		I	I					I	I						
*Lotus corniculatus var. tenuifolius*							I		I		III			III								I					I		IV	II								I	
*Isolepis cernua*					I																	I		II															
*Limonio gmelinii-Aeluropetum littoralis* (Bab. 1979) Gehu & Uslu 1989																																							
*Aeluropus littoralis*																I	V	II	I			I		III	I	III	III			I	II		II				I		
*Cressa cretica*																I	V	II	II	II	IV					I	I			I			I						
*Polypogono maritimi ssp. maritimi-Juncetum littoralis* Çakan, Düzenli & Karaömerlioğlu 2003																																							
*Juncus littoralis*										I	I	I		I						II		V					I	V	III	II	I						I		
*Phragmiti australis-Juncetum maritimi* Vural, Duman & al 1995																																							
*Juncus maritimus*				I					I											I			V	I	IV	I	II				I					I	I	I	
*Atriplici hastatae-Juncetum acuti* Çakan, Düzenli & Karaömerlioğlu 2003																																							
*Juncus acutus*																				I		I	I	V	I	I	I	I		II							I	I	
*Atriplex hastata*																							I	I			II			I							II		I
*Aster subulatus*									I					I										III	I	I	I	III		II									I
*Spergularia maritima*																		I						II			I												
*Arthrocnemo fruticosii-Tamaricetum tetragynae ass. nov.*																																							
*Tamarix tetragyna*																				I						V													
*Salicornio fragilis-Tamaricetum tetrandrae ass. nov.*																																							
*Tamarix tetrandra*																								I			V			I							I		
*Salicornia fragilis*																		I	I			I	II	I	II	II	III				I		II				I		
*Schoeno nigricantis-Saccharetum ravennae* Çakan, Düzenli & Karaömerlioğlu 2003																																							
*Saccharum ravennae*												II	II			I												V											
*Tamarix parviflora-Limonium angustifolium* society																																							
*Tamarix parviflora*																														V							I		
Phragmito-Magnocaricetea Klika in Klika & Novák 1941																																							
*Lythrum salicaria*												I																						I	II			I	I
*Lycopus europaeus*																																			III			I	I
*Bolboschoenus maritimus subsp maritimus*																											I											I	
*Cyperus longus*																																			I				
*Eleocharis palustris*																								I															
Nasturtio-Glycerietalia Piggn. 1954																																							
*Glycerio-Sparganion* Br. Bl. Et Sissingh in Boer 1942																																							
*Ludwigio stoloniferae-Nasturtietum officinalis ass. nov.*																																							
*Nasturtium officinale*																																		I	V			I	
*Ludwigia stolonifera*																																			IV			I	
*Phragmition communis* Schmale 1939																																							
*Paspalum paspalodes*																											I											III	II
*Cyperus serotinus*																							I																
*Ruppio cirrhosae-Schoenoplectetum litoralis ass. nov*																																							
*Schoenoplectus litoralis*																						I	I		II		I							II	II	V	II		II
*Ruppia cirrhosa*																																				III	I		
*Bolboscoeno maritimi var. cymos-Phragmitetum australis* Boridi &Balog 1970																																							
*Phragmites australis*		I		I		I			II	IV										IV	II	II	V	III	V	III	V		I	III	I			V	III	V	V	V	V
*Bolboschoenus maritimus var. cymosus*													I				V	I				III	III	II	III	II	III	I		II	I					III	IV	II	II
*Typha angustifolia-Juncellus laevigatus* society																																							
*Typha angustifolia*																								I	II					I				IV	III		I	V	
*Juncellus laevigatus*																						I		I												I		II	I
*Typha domingensis-Juncus pygmaeus* society																																							
*Typha domingensis*																																			I		I		V
*Juncus pygmaeus*				I	I		I		II	III												I		I		I	I	I	I		I	I	I						II
Potametea Klika in Klika & Novák 1941																																							
*Potamogeton panormitanus*																																			III	I	I		
*Potamogeton crispus*																																					I		
*Potamogetono pectinati-Ceratophylletum demersi* (Hild & Rehnelt 1965) Passarge 1995																																							
*Ceratophyllum demersum*																																		V	III			II	
*Potamogeton pectinatus*																																		V	III		I	II	
*Potamogeton nodosus*																																		II			I	II	
Molinio-Juncetea Br.-Bl. (1931) 1947																																							
Holoschoenetalia Br.-Bl. (1931) 1947																																							
*Salicornia europaea*	II																II	I		II		I	I	II		III	III		I	II	I		I				I		
*Molinio-Holoschoenion* Br.-Bl. (1931) 1947																																							
*Scirpoides holoschoenus*							I		II	III	III	I	II	III		IV						I						V	III	II							I		
*Eriantho-Schoenotum nigricantis* (Pign. 1953) Gehu 1984																																							
*Schoenus nigricans*												II		I		I												I	V	I									
*Plantago maritima*	IV						II					I		I		I				I		I		I		I			III										
Stellarietea mediae Tüxen, Lohmeyer & Preising ex von Rochow 1951																																							
*Digitaria sanguinalis*				I	I	II			II		I	II	II	I	V	I			I			I		II				I	II	I				I	I				I
*Anagallis arvensis var. caerulea*				III		I	I		II	II		I			II	I		II	IV		II					I						I	I						
*Lagurus ovatus*				III	I	II	III	III	II		V	III	III	III		V										I				I									
*Bromus tectorum*			I	III	I	I	I	I	I		III	I	III	I		I														II									
*Brassica tournefortii*				I		I			I		II	II	II	I		I														I									
*Crepis foetida subsp. commutata*				II		I	III						I			I								I				I		I									
*Senecio vulgaris*																		II	I		IV										I	I	I						
*Anagallis arvensis var. arvensis*				I			I		II						II			I													I								
*Panicum repens*																								I		I	I											I	
*Rhamnus oleoides subsp. graecus*											V	I		II		I																							
*Sonchus oleraceus*									I							I								I															
*Raphanus raphanistrum*															IV																								I
*Cardaria draba*																			I							I													
*Capsella bursa-pastoris*					I																																		
*Lamium amplexicaule*																I																							
Sisymbrietalia officinalis J. Tüxen in Lohmeyer & al. 1962 em. Rivas-Martínez, Báscones, T.E. Díaz, Fernández-González & Loidi 1991																																							
*Crepis vesicaria*				I	II	II	I		II		I				II	I		II	III		V	I		I		I					I	I	I						
*Geranium molle subsp. molle*						I						I						I								I	I												
*Hordeion leporini* Br.-Bl. in Br.-Bl., Gajewski, Wraber & Walas 1936 corr. O. Bolòs 1962																																							
*Plantago lagopus*							II		I		II			II	IV			I	V		V	I						II	I		I								
*Chrysanthemum coronarium*						I													I																				
*Laguro ovati-Bromion rigidi* Gahu et Gehu Franck 1985																																							
*Silene colorata*						I	II								IV						II																		
Saginetea maritimae Westhoff, Van Leeuwen & Adriani 1962																																							
*Bupleurum orientale*							I											I	I	I	II			II	I	I	I			II	I	I							
*Catapodium rigidum subsp. rigidum var. rigidum*				IV		I	IV	I	II	II	III	I	II			II												I		I									
*Crypsis faktorovskyi*												I										I				I				I									

## Data Availability

The data presented in this study are available upon request from the corresponding author.

## Appendix A

**Table A1:** Syntaxonomic units defined according to the main habitats and subgroups.

Habitats		Classes	Ordo	Alliance	Association
Aquatic Vegetation	The vegetation in the channel	Potametea Klika in Klika & Novák 1941	Utricularietalia Den Hartog & Segal 1964	*Ceratophyllion demersi* Den Hartog & Segal ex Passarge 1996	*Potamogetono pectinati-Ceratophylletum demersi* (Hild & Rehnelt 1965) Passarge 1995
		Phragmito-Magnocaricetea Klika in Klika & Novák 1941	Nasturtio-Glycerietalia Piggn. 1954	*Glycerio-Sparganion* Br. Bl. Et Sissingh in Boer 1942	*Ludwigio stoloniferae-Nasturtietum officinalis* ass. nov.
	The vegetation in the lake	Phragmito-Magnocaricetea Klika in Klika & Novák 1941	Phragmitetalia Koch 1926	*Phragmition communis* Schmale 1939	*Ruppio cirrhosae-Schoenoplectetum littoralis* ass. nov.
	Vegetation of the river and lake edges	Phragmito-Magnocaricetea Klika in Klika & Novák 1941	Phragmitetalia Koch 1926	*Phragmition communis* Schmale 1939	*Bolboschoeno maritimi* var. *cymos-Phragmitetum australis* Borhidi &Balogh 1970
Sand dune vegetation	Moving coastal sand dune vegetation	Ammophiletea Br.-Bl. & Tüxen ex Westhoff, Dijk, & Passchier 1946	Euphorbio-Ammophiletalia arundinaceae Br.-Bl. (1931) 1933 em. J.M. et J. Gehu 1988	*Euphorbio-Ammophilion arundinaceae* Br.-Bl. (1931) 1933 em. J.M. et J. Gehu 1988	*Cakilo maritimae-Zygophylletum albi* Çakan, Düzenli, & Karaömerlioğlu 2003
					*Cypero mucronati-Agropyretum juncei* Kühnholtz ex Br.-Bl. 1933
					*Eryngio maritimi-Pancratietum maritimi* Çakan, Düzenli, & Karaömerlioğlu 2003
					*Ambrosio maritimae-Pancratietum maritimi* ass. nov.
	Fixed or semi-shifting sand dune vegetation	Ammophiletea Br.-Bl. & Tüxen ex Westhoff, Dijk, & Passchier 1946	Euphorbio-Ammophiletalia arundinaceae Br.-Bl. (1931) 1933 em. J.M. et J. Gehu 1988	*Euphorbio-Ammophilion arundinaceae* Br.-Bl. (1931) 1933 em. J.M. et J. Gehu 1988	*Cypero capitati-Trachomitetum veneti* ssp. *sarmatiense* ass. nov.
					*Echio angustifolii-Ononidetum natrix* ssp. *hispanicae* ass. nov.
					*Parapholido incurvae-Thymelaeetum hirsutae* ass. nov.
	Dune scrub vegetation	Ammophiletea Br.-Bl. & Tüxen ex Westhoff, Dijk, & Passchier 1946	Euphorbio-Ammophiletalia arundinaceae Br.-Bl. (1931) 1933 em. J.M. et J. Gehu 1988	*Euphorbio-Ammophilion arundinaceae* Br.-Bl. (1931) 1933 em. J.M. et J. Gehu 1988	*Sorgho halepense* var. *halepense-Myrtetum communis* ssp. *communis* ass. nov.
					*Polygono equisetiformis-Viticetum agni-casti* ass. nov.
					*Ephedro campylopodae-Populetum euphraticae* ass. nov.
	Damaged sand dune vegetation	Ammophiletea Br.-Bl. & Tüxen ex Westhoff, Dijk, & Passchier 1946	Euphorbio-Ammophiletalia arundinaceae Br.-Bl. (1931) 1933 em. J.M. et J. Gehu 1988	*Euphorbio-Ammophilion arundinaceae* Br.-Bl. (1931) 1933 em. J.M. et J. Gehu 1988	*Urgino maritimi-Asphodeletum aestivi* ass. nov.
					*Verbasco sinuati-Sarcopoterietum spinosi* ass. nov.
Halophytic vegetation	Halophytic vegetation of the swamp	Salicornietea fruticosae Br.-Bl. & Tüxen ex A. & O. Bolòs 1950	Salicornietalia fruticosae Br.-Bl. 1933	*Salicornion fruticosae* Br.-Bl. 1933	*Limonio angustifolii-Halimionetum portulacoides* ass. nov.
					*Asterisco aquaticae*-*Plantaginetum coronopi* ssp. *commutati* ass. nov.
		Juncetea maritimi Br.-Bl. in Br.-Bl., Roussine & Nègre 1952	Juncetalia maritimi Br.-Bl. 1931 em. Julve 1992 ex 1993	*Juncion maritimi* Br.-Bl. 1931 em. Julve 1992 ex 1993	*Limonio gmelinii-Aeluropetum littoralis* (Bab. 1979) Gehu & Uslu 1989
	Halophytic vegetation of temporary ponds	Juncetea maritimi Br.-Bl. in Br.-Bl., Roussine & Nègre 1952	Juncetalia maritimi Br.-Bl. 1931 em. Julve 1992 ex 1993	*Juncion maritimi* Br.-Bl. 1931 em. Julve 1992 ex 1993	*Polypogono maritimi* ssp. *maritimi-Juncetum littoralis* Çakan, Düzenli, & Karaömerlioğlu 2003
					*Phragmiti australis-Juncetum maritimi* Vural, Duman, & al. 1995
					*Atriplici hastatae-Juncetum acuti* Çakan, Düzenli, & Karaömerlioğlu 2003
	Halophytic vegetation of temporary floodplain	Salicornietea fruticosae Br.-Bl. & Tüxen ex A. & O. Bolòs 1950	Salicornietalia fruticosae Br.-Bl. 1933	*Halocnemion strobilacei* Gehu & Costa 1984	*Tamaricetum smyrensis* Seçmen & Leblebici 1996
		Juncetea maritimi Br.-Bl. in Br.-Bl., Roussine & Nègre 1952	Juncetalia maritimi Br.-Bl. 1931 em. Julve 1992 ex 1993	*Juncion maritimi* Br.-Bl. 1931 em. Julve 1992 ex 1993	*Arthrocnemo fruticosii-Tamaricetum tetragynae* ass. nov.
					*Salicornio fragilis-Tamaricetum tetrandrae* ass. nov.
					*Schoeno nigricantis-Saccharetum ravennae* Çakan, Düzenli, & Karaömerlioğlu 2003
		Molinio-Juncetea Br.-Bl. (1931) 1947	Holoschoenetalia Br.-Bl. (1931) 1947	*Molinio-Holoschoenion* Br.-Bl. (1931) 1947	*Eriantho-Schoenotum nigricantis* (Pign. 1953) Gehu 1984
	Halophytic vegetation of terrestrial flatness	Salicornietea fruticosae Br.-Bl. & Tüxen ex A. & O. Bolòs 1950	Salicornietalia fruticosae Br.-Bl. 1933	*Salicornion fruticosae* Br.-Bl. 1933	*Salicornio europaeae-Arthrocnemetum fruticosum* Çakan, Düzenli, & Karaömerlioğlu 2003
				*Halocnemion strobilacei* Gehu & Costa 1984	*Arthrocnemo-Halocnemetum strobilaceii* Oberd 1952
